# ﻿Is *Ischnoderma benzoinum* a competitor or contributor to *Heterobasidion annosum* decomposition of pine and spruce wood? A comparison to *Phlebiopsis gigantea*

**DOI:** 10.3897/imafungus.16.152556

**Published:** 2025-09-09

**Authors:** Andrzej Szczepkowski, Leszek Bolibok, Zbigniew Sierota

**Affiliations:** 1 Department of Forest Protection, Institute of Forest Sciences, Warsaw University of Life Sciences-SGGW, Nowoursynowska 159, 02-776 Warszawa, Poland; 2 Department of Silviculture, Institute of Forest Sciences, Warsaw University of Life Sciences-SGGW, Nowoursynowska 159, 02-776 Warszawa, Poland; 3 Department of Forestry and Forest Ecology, Faculty of Agriculture and Forestry, Warmia and Mazury University in Olsztyn, Pl. Łódzki 2, 10-721 Olsztyn, Poland

**Keywords:** Antagonism, *
Basidiomycota
*, dual cultures, linear growth, Norway spruce, Scots pine, wood decay fungi

## Abstract

The ecological role of *Ischnoderma benzoinum* in conifer stands remains poorly understood, particularly with regard to its potential to compete with the root rot pathogen *Heterobasidion annosum*. This study investigated the growth dynamics, wood decay capacity, and competitive interactions of two *I. benzoinum* isolates (originating from *Pinus sylvestris* and *Picea abies*) with two *H. annosum* isolates from the same host species, and with the biocontrol fungus *Phlebiopsis gigantea*. Laboratory assays involved growth on malt extract agar for 14 days, and single and dual inoculations on pine and spruce wood for 60 and 120 days. Both *I. benzoinum* isolates exhibited growth rates on medium comparable to those of *H. annosum*. On spruce wood, *I. benzoinum* caused the greatest mass loss, whereas *H. annosum* dominated on pine. In most dual cultures, wood mass loss was similar to that in single cultures; however, in the pairing of the spruce-derived *I. benzoinum* with *P. gigantea*, decay was significantly reduced, indicating antagonistic interaction. Competitive outcomes in dual cultures varied markedly. The pine-derived *I. benzoinum* strongly suppressed the pine-derived *H. annosum*, whereas the spruce-derived *H. annosum* consistently outcompeted both *I. benzoinum* isolates. *P. gigantea* strongly inhibited *I. benzoinum* mycelial growth and frequently reduced its decay activity. These findings demonstrate that *I. benzoinum* can function either as a strong competitor or as a co-colonizer with *H. annosum*, with interaction outcomes determined by isolate origin and host tree species. The capacity of *I. benzoinum* to match *H. annosum* in wood decay efficiency, particularly on spruce, suggests that it may influence disease progression and nutrient cycling in coniferous forests. This work advances understanding of fungal community dynamics in wood decomposition and highlights the need for further ecological and molecular studies to clarify the role of *I. benzoinum* in forest health and management.

## ﻿Introduction

### ﻿Root rot pathogens in managed forests

*Heterobasidion annosum* (Fr.) Bref. s.l. is considered one of the most damaging fungi affecting tree roots and trunks, especially in the northern hemisphere (Laine 1976; Garbelotto and Gonthier 2013; Gonthier and Thor 2013). Root diseases caused by *H. annosum* result in reduced stand growth, stand self-thinning, tree death, and often the collapse of entire stands, with millions of euros in annual economic losses (Ferguson et al. 2003; Oliva and Colinas 2010; Gori et al. 2013; Sierota 2013; Krisans et al. 2020; Oszako et al. 2023). The presence of this pathogen is manifested by significant changes in the age, spatial and species structure of the entire stand, as well as reduced resistance to pests and to toppling by wind and snow (Giordano et al. 2012; Honkaniemi et al. 2017; Sierota et al. 2019). Tree death, the formation of stand gaps exposing the forest floor to allow natural regeneration in monocultures, especially those on afforested land in the United Kingdom and Central and Eastern Europe, can have significant and in some situations positive effects on forest ecosystem (Holah et al. 1997; Brockway and Outcalt 1998; Hansen and Goheen 2000; Manion 2003). Thus, although pathogens can have unwanted effects in commercial stands, they also can have desirable effects in shaping forest ecosystems (Winder and Shamoun 2006; Lonsdale et al. 2008; Kubiak et al. 2017; Sierota and Miścicki 2022; Kuryło et al. 2024).

*Ischnoderma benzoinum* (Wahlenb.) P. Karst., from the group of white rot-forming basidiomycetes, is increasingly found in many countries (e.g., Belgium, Finland, Sweden, France, Russia), including in Polish coniferous forests (Hintikka 1993; Lapadatescu et al. 1997; Vandekerkhove et al. 2011; Teplyakova et al. 2012; Carlsson 2014; Kujawa et al. 2024). To date, this fungus and the etiologically similar *I. resinosum* (Schrad.) P. Karst. have mainly been observed in old stands or spruce stands (Domański et al. 1973; David et al. 1983). The morphology of the basidiomata and the way the mycelium is embedded in the wood are very similar to *H. annosum*, *Heterobasidion parviporum* Niemelä & Korhonen, and *Heterobasidion abietinum* Niemelä & Korhonen, often leading to inaccurate monitoring assessments and reducing the reliability of information on forest condition and biodiversity (Dennis and Watling 1983; Allen et al. 2022).

*Ischnoderma* is isolated within the *Polyporales* and forms its own monotypic family, *Ischnodermaceae* (Justo et al. 2017). *I. benzoinum* is found in coniferous forests throughout Europe, northern Asia and North America (Breitenbach and Kränzlin 1986; Ryvarden and Gilbertson 1993; Dai 2012). It has been described as fairly common in native forests and in areas impacted by humans (Niemelä and Kotiranta 1986; Dai 2012; Korhonen et al. 2012; Kujawa et al. 2018; Bernicchia and Gorjón 2020; Wołkowycki 2022). However, some authors state that it prefers extensively used forests, especially in mountainous areas and in older forests (Domański et al. 1973; Niemelä 2013), which is probably why it is a Red Listed fungus in some countries, including Poland (Wojewoda and Ławrynowicz 2006).

**Figure 1.**
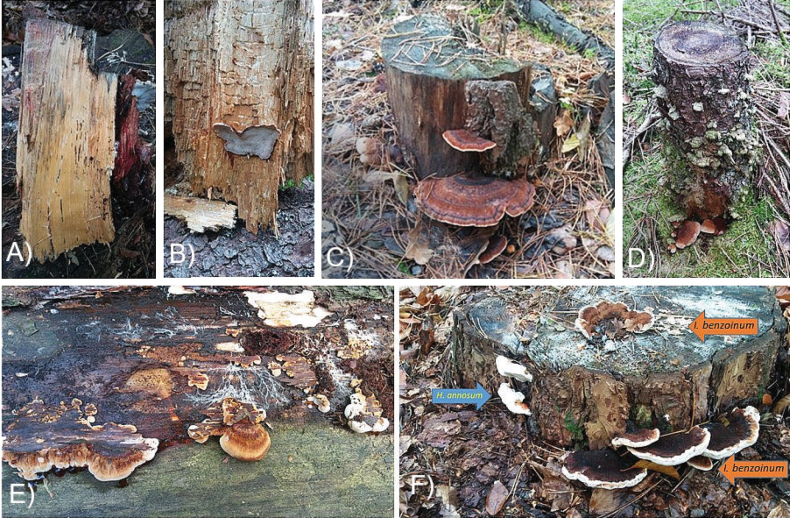
*Ischnoderma benzoinum* – a fragment of pine stump with visible wood rot (**A**), a fragment of decayed spruce wood with basidiomata (**B**), basidiomata on a pine stump (**C**) and on a spruce stump (**D**), basidiomata and white mycelium with rhizomorphs after removal of the bark on a pine log (**E**), a pine stump with basidiomata of *I. benzoinum* (right and upper side of stump) and *Heterobasidion annosum* (left) (**F**) (all photos by A. Szczepkowski).

*Ischnoderma benzoinum* causes white rot in dead conifers (Fig. 1A, B). The decayed wood has a strong aniseed odor and appears yellowish, ranging from thread-like to spongy in texture (Ryvarden and Gilbertson 1993). According to Domański et al. (1973), it causes intensively developing white rot of wood. It grows on dead standing or downed conifers, on branches and stumps (Fig. 1) (Breitenbach and Kränzlin 1986; Dai 2012; Bernicchia and Gorjón 2020), especially frequently on *Picea*, but also *Abies*, *Cedrus*, *Larix*, *Pinus*, *Pseudotsuga*, *Thuja*, but very rarely on deciduous species such as *Alnus*, *Fagus*, and *Prunus* (Kotlaba 1984; Ryvarden and Gilbertson 1993; Dai 2012; Ryvarden et al. 2017; Rivoire 2020). The basidiomata of *I. benzoinum* (Fig. 1C–F) were described in detail by Ryvarden and Gilbertson (1993), Bernicchia (2005), Niemelä (2013), Ryvarden et al. (2017), Bernicchia and Gorjón (2020) and Rivoire (2020).

### ﻿Antagonism and competition between root rot fungi

The similarity of the mode of colonization of conifer roots and trunks by *Ischnoderma*, the similarity of wood decomposition, and the similarity of the basidiomata with the fungus *Heterobasidion*, indicate the potential pathogenicity of *Ischnoderma*, especially in commercial stands on post-agricultural soils (Sierota 1997). Such a hypothesis immediately suggests the possibility of biologically controlling *Ischnoderma*, especially using the competing fungus *Phlebiopsis gigantea* (Fr.) Jülich. *P. gigantea* is used for biological control and has been described as one of the most effective methods for controlling root rot (Pratt et al. 2000; Vainio et al. 2001; Webber and Thorpe 2003; Berglund and Rönnberg 2004; Sierota et al. 2007; Brûna et al. 2020). However, global climate change and local weather anomalies have been causing new behaviors of root pathogens and associated organisms (Kubiak et al. 2017; Singh et al. 2018; Venäläinen et al. 2020; Boczoń et al. 2021; Sierota et al. 2022). This indicates the high adaptive plasticity of fungi, bacteria, nematodes, mites, and viruses, and the as yet not fully understood relationships between them. Research attention has been focused on fungi and viruses accompanying *Heterobasidion* spp., as well as on bacteria and fungi developing in rhizomorphs of *Armillaria*, new invasive species of pathogens, and differential activity of different fungal species isolates (Gonthier et al. 2014; Sierota et al. 2015; Tomalak 2017; Przemieniecki et al. 2021; Szczepkowski et al. 2022).

Recent studies of fungal species coexisting on the same substrate described their competition and antagonism (Thambugala et al. 2020; Cui et al. 2023; Hınçal and Yalçın 2023). Żółciak et al. (2016) described possible competition between *Antrodia gossypium* (Speg.) Ryvarden, *P. gigantea* and *H. parviporum* in spruce wood. Burņeviča et al. (2022) described the role of some Latvian isolates of *Bjerkandera adusta* (Willd.) P. Karst. and *Sistotrema brinkmannii* (Bres.) J. Erikss. in controlling *Heterobasidion* infections, similar to Piri and Vainio (2024), who described control by *Stereum sanguinolentum* (Alb. & Schwein.) Fr. and *Amylostereum areolatum* (Chaillet ex Fr.) Boidin. The activity of *Trichoderma* species and their volatile and non-volatile metabolites against pathogens of plants and other harmful microorganism is well known (Monaco et al. 1991; Raut et al. 2014; Meena et al. 2017; Kumar et al. 2019), as are those of many other microbial antagonists (Heydari and Pessarakli 2010). However, La Porta et al. (2001) found that *Trichoderma viride* Pers. poorly controlled *H. annosum*.

Fungal relationships in dual cultures can help explain the interactions of mycelia on the same food base, even when these interactions occur under non-natural laboratory conditions (Badham 1991; Holmer and Stenlid 1993; Velázquez-Cedeño et al. 2004). For example, Nawrot-Chorabik (2013) evaluated interactions between fungi in dual cultures on pine callus and found an inhibitory effect of *Phacidium lacerum* Fr. towards *Gremmeniella abietina* (Lagerb.) M. Morelet and *Anthostomella formosa* Kirschst. Osmenda and Nawrot-Chorabik (2024) described interesting interactions in dual cultures of fungal species colonizing *Pinus sylvestris* trees and concluded that *Ophiostoma minus* (Hedgc.) Syd. & P. Syd. and *Trichaptum fuscoviolaceum* (Ehrenb.) Ryvarden can greatly restrict the growth of other fungi, e.g., *Skeletocutis* sp. and *Armillaria ostoyae* (Romagn.) Herink. The main interactions between microorganisms occur through enzymes produced and induced in response to the presence of a partner. Enzyme activity blocks both the transcription and translation of plant resistance genes (mRNA synthesis and protein expression) and the dissolution of host cell membranes (Vincenti et al. 1996; Adams 2004).

The ability of *I. benzoinum* to produce enzymes has not been studied in detail, however *I. benzoinum* produces laccase and manganese peroxidase, allowing it to decolorize synthetic dyes (Kinnunen et al. 2017; Eichlerová and Baldrian 2020). *I. benzoinum* degrades L-phenylalanine, enabling microbial production of aromatic benzaldehydes (Lomascolo et al. 1999). Zorn et al. (2003) pointed out the ability of *I. benzoinum* to cleave beta-carotene into aromatic compounds. Anke et al. (1982) found that the antibiotic 1-hydroxy-2-nonyn-4-one isolated from submerged cultures of different strains of *I. benzoinum*, was highly inhibitory of yeasts and filamentous fungi at 1–5 µg/ml concentrations. Kirk and Moore (1972) found that a related fungus, *I. resinosum*, utilizes 35% of the lignin in the degradation of 10–12% of the dry mass of wood. Kokol et al. (2007) reported that *I. resinosum* produces extracellular laccase (p-diphenol oxygen oxidoreductase) and manganese peroxidase (MnP), potent ligninolytic enzymes, with maximum activity after 10–14 days. Thus, *Ischnoderma* species produce strong laccase-group enzymes, similarly to *H. annosum* (Maijala 2000; Asiegbu et al. 2004; Kuo et al. 2015; Aza et al. 2023). *Ischnoderma* spp. may therefore be involved in the colonization of wood, roots or stumps by active decomposition of cell walls and, together with *H. annosum*, may threaten tree health. The ability to produce these enzymes indicates the enzymatic activity of pathogens, e.g., guaiacol (2-methoxyphenol), which turns from colorless to cherry-brown tetraguaiacol under the influence of laccase (for instance, phenoloxidase) (Havličkova and Rypaček 1957; Karla et al. 2013; Abd El Monssef et al. 2016; Raner and Hess 2023).

The characteristics of mycelial growth, the resulting wood decay, and the interactions between *I. benzoinum* and the pathogens *H. annosum* s.l. and the increasingly widespread *P. gigantea* are not yet fully understood (Khanso and Khansob 1985). Since the above species can occupy the same ecological niches (stumps and roots), we conducted laboratory studies to understand the modes of development and interactions between these fungi.

We are not aware of descriptions of reactions in dual cultures of *I. benzoinum*, *H. annosum* and a competitor of *H. annosum*, *P. gigantea*, especially when grown using inoculum obtained from pine and spruce wood. The aim of the present study was to determine the growth rates of isolates from the three species (*I. benzoinum*, *H. annosum*, and *P. gigantea*) in pure cultures separately and in double cultures together, as well as to determine the degree of decomposition of wood seeded with these isolates. We also evaluated the behavior of fungal isolates originating from pine versus spruce. We hypothesized that mycelium of *I. benzoinum* would exhibit similar growth activity and decomposition efficiency to that of the pathogen *H. annosum*, whether on MEA or in wood samples, regardless of the source of the isolate, with concomitant mycelial growth. Our aim in this direct contact between these fungi in a controlled study was to elucidate the relationships that exist under natural conditions, which are difficult to assess since it is already known that their mode of substrate colonization may vary. It is known that *H. annosum* infects both roots as well as the side of stumps, while *P. gigantea* infection is only from the side of the stump (Sierota 1995; Pratt et al. 2000; Gaitnieks et al. 2020), while it is unclear whether *I. benzoinum* colonizes roots. Understanding the mutual reactions and the degree of decomposition of wood colonized by these fungi will allow us to determine the degree of competitiveness of *I. benzoinum* against *H. annosum*, as well as their interaction in the presence of *P. gigantea* in the same food base.

**Figure 2.**
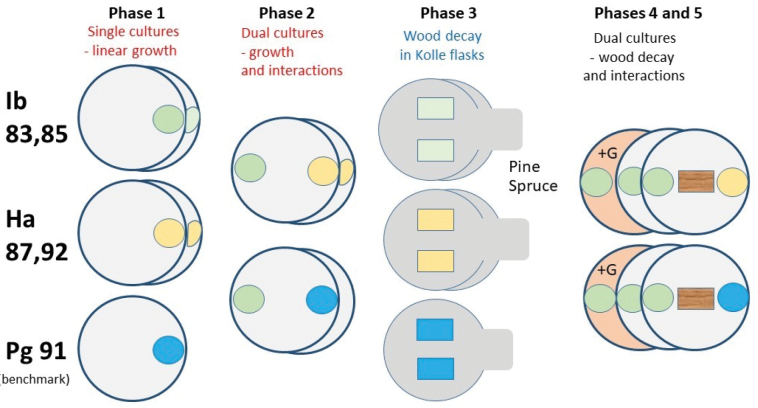
Scheme of the laboratory tests with five isolates (Ib83, Ib85, Ha87, Ha92, Pg91), two wood species (Scots pine, Norway spruce) on MEA medium and with MEA and guaiacol (+G). Colors indicate fungal isolates.

In order to address the goals of this study, the experiment was divided into five phases (Fig. 2):

1. evaluation of the linear growth of five isolates tested on MEA – *Ischnoderma benzoinum* from two isolates (Ib83 and Ib85), *Heterobasidion annosum* from two isolates (Ha87 and Ha92), and one isolate of *Phlebiopsis gigantea* (Pg91);
2. evaluation of the growth and type of mycelial interaction in dual cultures on MEA;
3. evaluation of wood dry matter loss in Scots pine and Norway spruce samples separately, caused by isolates tested on MEA;
4. evaluation of dry wood mass loss in dual cultures; and
5. evaluation of the interactions between fungi colonizing wood samples.

## ﻿Materials and methods

### ﻿Isolates

Isolates came from the collection of pure cultures of the Department of Forest Protection of the Warsaw University of Life Sciences (WAMLCK) (Table 1).

**Table 1.** Characteristics of the isolates of three fungal species used in the study.

Isolate code from WAMLCK	Fungus	Host(species/part)	Locality and coordinates	Reference sequences for NCBI				Number of sequences ITS	Number of sequences EF
				ITS1	Identity (%)	TEF 1	Identity (%)		
83	*Ischnoderma benzoinum*	*Picea abies* (L.) H. Karst. at the trunk of the windthrown tree	Romincka Forest, Gołdap FD, Dziki Kąt 70 (NE Poland) 54.34°N, 22.59°E	MH861247	99.83	ON424839	98.83	PQ518568	PQ216352
85	*Ischnoderma benzoinum*	*Pinus sylvestris* L. stump	^†^LZD Rogów, Lipce Reymont. 36 d (C Poland) 51.91°N, 19.90°E	MH861247	99.83	ON424839	98.06	PQ518569	PQ216354
87	*Heterobasidion annosum*	*Picea abies* lying log	Romincka Forest, Gołdap FD, Boczki 144 (NE Poland) 54.33°N, 22.51°E	MH931274	99.67	JN657498	92.37	PQ518570	PQ216353
92	*Heterobasidion annosum*	*Pinus sylvestris* stump	LZD Rogów, Lipce Reymont. 36 d (C Poland) 51.91°N, 19.90°E	MK95162	98.80	JN657498	96.59	PQ518572	PQ216355
91	*Phlebiopsis gigantea*	*Pinus sylvestris* the base of a dead pine trunk	LZD Rogów, Lipce Reymont. 36 d (C Poland) 51.91°N, 19.90°E	MT386381	97.17	MZ913623	97.10	PQ518571	PQ216356

^†^LZD – The Forest Experimental Station of WULS-SGGW in Rogów.

### ﻿Media (Phases 1 and 2)

**Figure 3.**
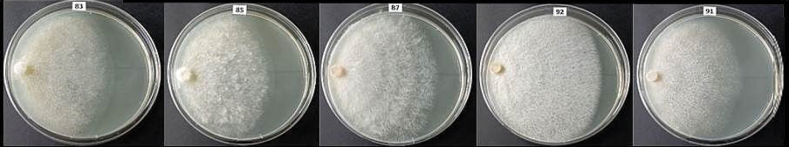
Growth of pure cultures of five isolates after 12 days on MEA – (from left to right): tested *Ischnoderma benzoinum* isolates 83 and 85, and testing *Heterobasidion annosum* isolates 87 and 92, and *Phlebiopsis gigantea* isolate 91.

The growth rates of five isolates, examined in both single and dual cultures, was evaluated on MEA medium (Carl Roth, Germany) in 13 cm (Fig. 3) and 8 cm Petri dishes; 50 ml of medium was poured onto each 13 cm plate, and 20 ml onto 8 cm plates. After the agar solidified, a mycelial plug of standardized diameter of 1 cm from each fungal species was inoculated onto the side wall of the Petri dish. This step was repeated for each species used in the study. Dishes were sealed with parafilm and incubated at 22 ± 2 °C for 14 days in the dark. Radial growth of the cultures was measured using a graph paper strip with an accuracy of 1 mm every 48 hours over 14 days. Growth is the average of five replicates of each isolate, in single and dual cultures.

### ﻿Wood decay assessment (Phases 3 and 4)

Wood samples were taken from the sapwood of healthy trees (no defoliation, no root rot in the stands) in stands of mature Scots pine (Ciechanów Forest District – C Poland) or Norway spruce (Starachowice Forest District – CS Poland). Slats were made from the lower part of the trunks, and corresponding strips were cut, from which wood samples in blocks of 1.5 × 2.5 × 5.0 cm were produced. Sierota et al. (2016) assumed an average wood density of 376 kg/m^3^ as the limit between the minimum and maximum density of spruce wood – here, 514 kg/m^3^ (539 kg/m^3^ for pine samples). Considering this criterion, it can be concluded that all wood blocks of both species had higher wood density and thus a higher proportion of latewood in the annual increment than of early wood.

Decay tests using single cultures were carried out on 200 wood samples (100 samples of each tree species, including 100 samples examined after each of 60 and 120 days and 40 samples for each of the five fungal isolates). The ability of the fungi to decompose wood was determined based on dry mass loss of wood samples after 60 and 120 days. Test procedures were according to Polish standards PN-EN 350 (2018) and PN-EN-113 (2022), as well as Szczepkowski et al. (2021) and Marciszewska et al. (2024). In brief, each wood sample was measured with an accuracy of 0.01 mm for physical dimensions and with an accuracy of 0.01 g for weight after conditioning in air under laboratory conditions. For each tree species, the moisture content of six additional wood samples was calculated after drying to constant weight at 103 ± 2 °C after first equilibrating to laboratory conditions. Sample mass loss (%) due to fungal decomposition was determined based on dry mass loss. Wood samples used in decay tests were not oven-dried prior to being used in the experiments.

Before the decay test, wood samples, which had similar wood density and dimensions, were sterilized twice at intervals of 24 hours in an autoclave at 121 °C and 0.12 MPa pressure for 20 and 10 minutes respectively. They were then soaked in sterile distilled water for approximately one hour. After soaking, two wood samples were placed in Kolle flasks with approximately three-week-old cultures of the selected fungal species in 50 ml of 2% MEA medium (Carl Roth, Germany). After 60 or 120 days of incubation at 22 ± 2 °C and 70 ± 5% relative humidity, one of two samples was removed, freed from the surface mycelium and weighed. Wood samples were then dried at 103 ± 2 °C until they reached a constant mass, i.e., when weight measured at 4-hour intervals was within 0.05 g, after which they were weighed again and that value was used to calculate wood mass loss. The same procedures were carried out for the second wood sample in each flask after 120 days.

**Figure 4.**
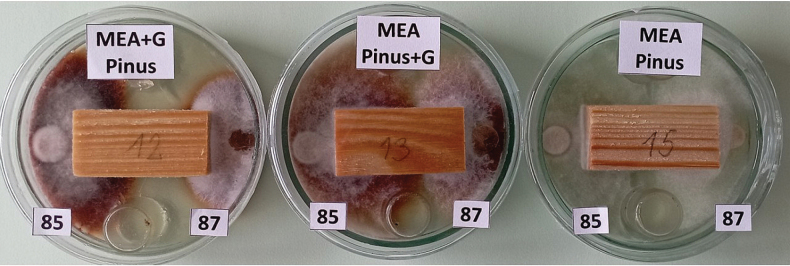
Visualization of the activity of isolates after nine days of culture growth on media with guaiacol; here: mycelial growth of *Ischnoderma* Ib85 and *Heterobasidion* Ha87 isolates on pine wood samples on MEA medium with guaiacol (left), on MEA with guaiacol-saturated wood (center) and for comparison – on MEA medium (right).

Dual fungal culture comparisons were performed in seven combinations: isolate 83 versus 85, 85 v. 87, 85 v. 91, 85 v. 92, 83 v. 87, 83 v. 91 and 83 v. 92. Each of the seven dual isolate comparisons of wood decomposition was represented by 3 pine blocks and 3 spruce blocks (42 blocks in total), with one uninoculated block for each wood species treated as a control and placed on MEA. To visualize the enzymatic effect of phenol oxidase activity of certain isolates, some blocks 1) were soaked in guaiacol (0.2 ml guaiacol per 0.5 l medium) and put on MEA (wood+G, MEA), 2) were soaked with sterile water and placed on MEA with guaiacol (0.5 l distilled water plus 0.2 ml guaiacol) (MEA+G, wood), and 3) were similarly soaked with water (Fig. 4). Since the study by Havličkova and Rypaček (1957), it has been established that *H. annosum* can decolorize guaiacol in media (Johansson et al. 1999; Kalra et al. 2013; Abd El Monssef et al. 2016). Similarly, it is known that *I. resinosum* produces active ligninolytic enzymes and decolorizes some dyes in liquid cultures (Sandes et al. 2023). This capability has been used in testing mycelial growth on wood samples in dual cultures as an additional visual assessment indicating phenol oxidase activity based on color intensity of the medium on both the front and back of the plates. The presence of guaiacol and the color reaction facilitated, therefore, the assessment of the relationship between the two partners.

Twenty milliliters of MEA (or MEA medium with guaiacol) was poured into 9.0 cm diameter Petri dishes, then two 2-cm-high cylinders and a wood block were placed directly on the medium. A 1 cm diameter inoculum sample (mycelium with agar) was placed 0.5 cm from the block adjacent to the wood cross-sectional surface, with one isolate on one side and another isolate on the other side (Fig. 4). The fungal inoculum and wood blocks were placed into Petri dishes on the same day. The incubation period in this experiment was 60 days. Wood loss in dual cultures was tested on 24 blocks with the combinations listed in Table 6. The weighing and mass calculation procedures were the same as for the single isolate wood decomposition evaluations. Mean weight loss was averaged from the samples of each tree species. In this experiment we visualized the enzymatic effect of phenol oxidase activity of certain isolates, by: 1) soaking some blocks in guaiacol (0.2 ml guaiacol per 0.5 l medium) and placing on MEA (wood+G, MEA), 2) soaking some blocks with sterile distilled water and placed on MEA with guaiacol (0.5 l distilled water plus 0.2 ml guaiacol) (MEA+G, wood), and 3) soaking blocks only with sterile distilled water (Fig. 4).

### ﻿Assessment of relations in dual cultures in dishes and on wood (Phases 2 and 5)

Agar blocks (Ø 1.0 cm) overgrown with a pure culture of the tested *Ischnoderma* isolates were placed on a sterile MEA Petri plate (Ø 8.0 cm), and similar agar blocks of *H. annosum* and *P. gigantea* were placed on the same plate at a distance of 6 cm apart. There were five replicate Petri plates for each variant, which were incubated in the dark at 22 ± 2 °C. Inhibition between isolates was assessed after 14 days.

**Figure 5.**
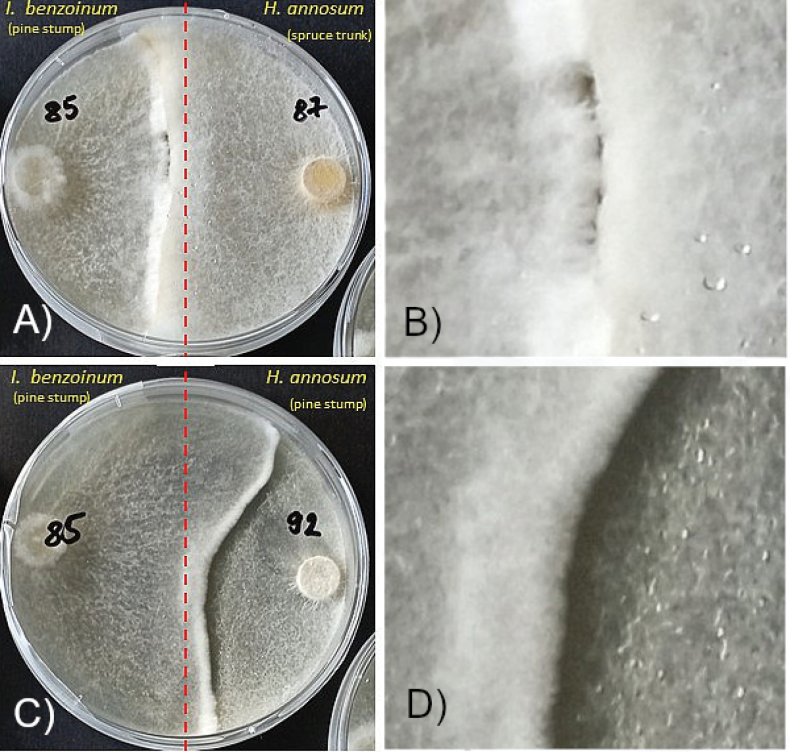
Examples of growth of *Ischnoderma* isolate Ib85 and two *Heterobasidion* isolates: Ib85 versus Ha87 – reaction type: loser L (**A, B**) and Ib85 versus Ha92 – reaction type: winner W (**C, D**) after 14 days.

Fungal isolate activity (*I. benzoinum* 85 and 83 and *P. gigantea* 91) against *H. annosum* isolates 87 and 92 was evaluated for all isolate combinations in 8 cm Petri dishes after 14 days and 6 weeks, using methods modified from Holdenrieder (1984) and Holdenrieder and Greig (1998). Interaction type scoring was performed, where: W (Winner) – the tested fungus *I. benzoinum* isolate overgrows *H. annosum* or there is a mycelium-free zone of inhibition between the fungi; D (Draw) – *I. benzoinum* forms a mycelium-free zone of inhibition with only one isolate of *H. annosum* or the boundary is less visible; L (Loser) – the mycelium of *H. annosum* overgrows the tested *I. benzoinum* mycelium (Fig. 5). For the dual confrontation, we used a visual binocular assessment of mycelial growth and demarcation area on MEA and on wood (as W, D, L). We evaluated the 6 samples without taking into account the wood species, although this might have influenced the development of aerial mycelium.

### ﻿Statistics

In the first phase of the experiment, the growth of five isolates of fungi on MEA were compared using a general additive model (GAM) to represent the nonlinear dependence between time from the onset of the experiment and the maximum extent (distance) of mycelium growth. Each isolate was grown on five dishes. Growth extent was measured in each dish of each isolate on seven dates. Measurements from one dish are probably more similar than those from other dishes, which violates the assumption of sample independence. To address this, a mixed-effect GAM model was used with dish ID as the random effect. To build the model, we used the gam function from the mgcv library (Wood 2011). In this implementation, the random effect is represented by an additional smoothing function in the model. Because extent measurements have strictly positive values and predicted values also should be positive, the gamma distribution was used in the model. The mathematical formula of the model is presented in eq. (1).

*log* (*μ_i_*) = *β*_0_ + *β*_1_ ∙ *isolate_i_* + *f_isolatei_* (*days_i_*) + *b_dishi_* (1)

where: *log* (*μ_i_*) – indicates that the model assumes a gamma distribution for *extent_i_ Gamma* (*μ_i_*) with log link and *extent_i_* is the outcome for observation *i*.

*β*_1_ ∙ *isolate_i_* – represents the fixed effect and *isolate* is a categorical variable with 5 levels corresponding to the isolate tested in the experiment

*f_isolatei_* (*days_i_*) – indicates smoothing terms for the effect of days, a thin plate spline with 5 basic functions which vary by *isolate*.

*b_dishi_* represents random effect *b_j_ N* (0,σ^2^_*dish*_), where *b_dishi_* is the random intercept for the dish

In the second phase of the experiment, using dual cultures on MEA, the small number of samples caused us to use Fisher’s exact test (Fisher 1935) to check for significant relationships between isolate type (categorical variable with five levels) and the confrontation outcome (categorical variable with three levels).

In the third phase of the experiment, a generalized linear model was used to describe the influence of different variables on percent dry matter loss of wood samples exposed to different fungal isolates. Although measurements were taken at two points in time, there was no need to implement a mixed-effect model. Because measurements of wood dry weight destroy the fungal isolates, time sequence estimates of wood decomposition were made on different wood samples. In the initial form of the model, four explanatory variables were added (tree species, isolate, sample wood density, and time). During model-building, interactions between variables were also tested. The final model included only two interaction terms based on statistical importance, the interaction between tree species and isolate and the interaction between isolate and time. Multiple comparisons of mean dry matter loss caused by particular isolates and the identification of homogenous groups were made using the emmeans library (Lenth 2024).

In phase four of the experiment, fungal antagonism was evaluated by comparing dry matter loss obtained in the third phase of the experiment for single-fungi decomposition of wood samples with dry matter loss in dual cultures. The assumption was that if there is no interference between isolates, then dry matter loss in dual culture should equal the average dry matter loss caused by the same isolates in wood samples tested alone. Statistically significant differences between expected and observed values of dry matter loss in dual cultures can be interpreted in two ways. If dry matter loss is larger in dual culture, it could indicate a stimulatory effect between isolates, and if dry matter loss is smaller, it could suggest an inhibitory effect. Ten single-culture dry matter loss values were obtained for each isolate. For example, for isolates Ib85 and Ha92, the 10 pairs of readings were randomly connected to calculate 10 mean values. During testing, 10 readings of expected dry matter loss were compared with three readings from dual cultures of a particular pair of isolates. The unequal number of measurements between the two culture types resulted from the loss of some dual cultures, and the experiment could not be repeated. Due to the challenge of confirming the normality of the distribution with a small sample size, readings were compared using the Mann-Whitney U test, although unequal sample sizes reduce, to some extent, the statistical power of this test and make results more sensitive to outliers in the smaller group.

The fifth phase of the experiment compared wood dry matter loss in dual cultures to determine whether there were significant relationships between isolates (categorical variable with five levels) in confrontation with *H. annosum* (categorical variable with three levels). Fisher’s exact test was used due to small sample size. The same test was used to evaluate the independence of the outcome of isolate confrontations and substrate type.

All calculations were made using an R statistical environment (R Core Team 2024).

## ﻿Results

### ﻿Linear growth in single cultures (Phase 1)

**Figure 6.**
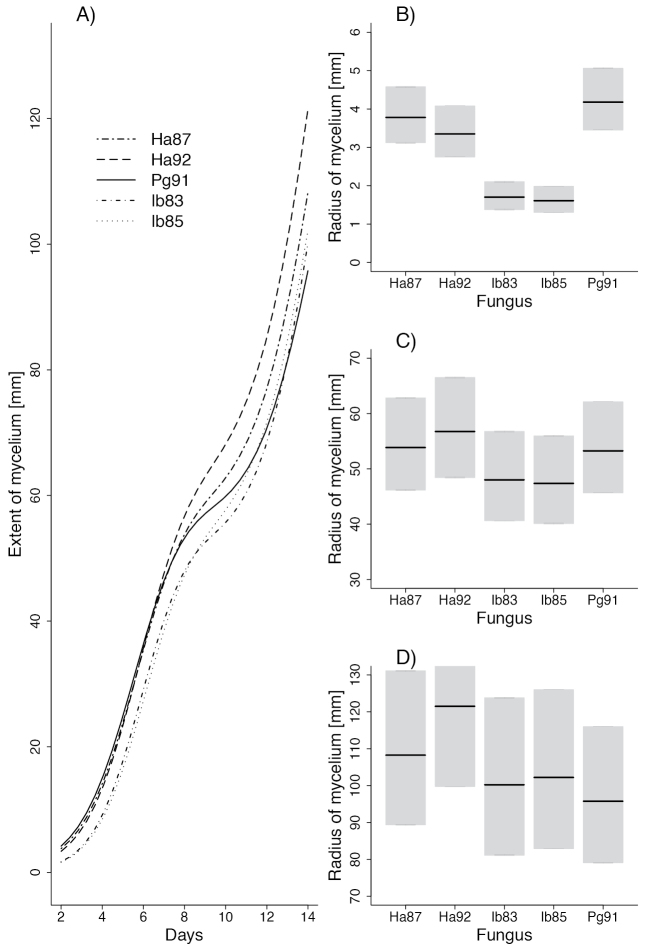
Average mycelial radius of the tested isolates at one-day intervals (**A**) and mean diameter of the cultures after 2 (**B**), 8 (**C**) and 14 days (**D**) on MEA in Petri dishes 13 cm in diameter

The model describing linear mycelium growth of fungal isolates is presented in Table 2. The smoothing function, represented as a random effect (s(dish.id), significantly improved the model (p < 0.001). Also, the nonlinear dependence of mycelia growth with time was confirmed for all isolates (e.g., s (days): Ha87 p-value < 0.001). In R, the first level of a factor (in our case the variable was isolate Ha87) is used as a reference level for comparison with other levels (isolates). Other levels are shown as differences in effect compared to the reference level. Testing indicates that only isolates Ib83 and Ib85 grew significantly differently than Ha87. Fig. 6 presents predictions from the model described above. Panel A shows the curvilinear dependence between isolate growth and time between days 2 and 14. Only at the beginning of observations were the confidence intervals for different isolates distinctly separated. Panels B–D show comparisons of mean mycelial extension of isolates (with confidence intervals in grey) after 2, 8 and 14 days. Only on day 2 are confidence intervals for isolates visibly separated, suggesting that isolates Ib83 and Ib85 at the beginning of the experiment grew distinctly more slowly than other isolates, but their later rate of growth was equivalent to other isolates.

**Table 2.** Generalized additive mixed models analyzing the effects of the day of the experiment fitted with spline (s) on the extent of mycelium of five fungi isolates.

Predictors	Extent of mycelium			
	Estimates	CI	Statistic	p
Intercept	33.86	29.47–38.90	50.16	<0.001
Ha92	1.03	0.84–1.25	0.25	0.800
Ib83	0.76	0.63–0.93	-2.75	0.007
Ib85	0.75	0.62–0.92	-2.85	0.005
Pg91	0.99	0.81–1.20	-0.10	0.919
s(days):Ha87			411.48	<0.001
s(days):Ha92			486.92	<0.001
s(days):Ib83			633.36	<0.001
s(days):Ib85			657.66	<0.001
s(days):Pg91			352.48	<0.001
s(dish.Id)			5.84	<0.001
Observations	175			
R2	0.974			

**Figure 7.**
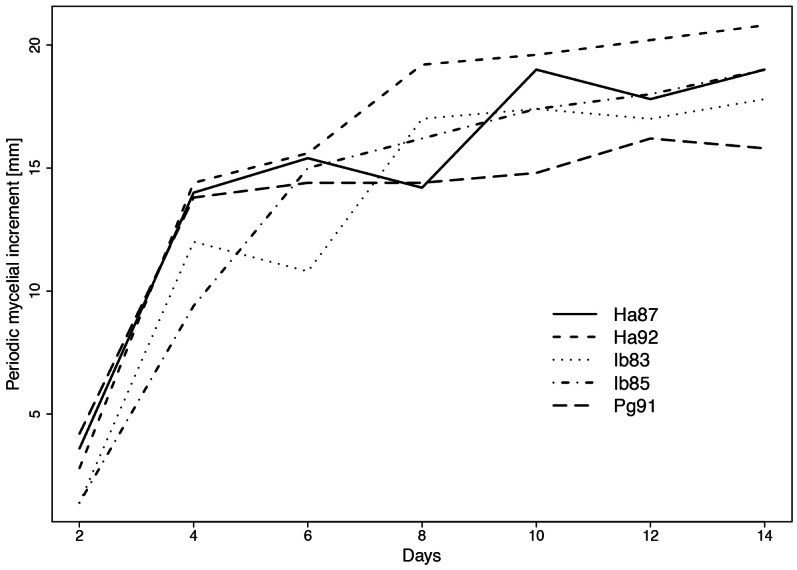
Average mycelial growth of *Heterobasidion annosum* (Ha87 and Ha92), *Ischnoderma benzoinum* (Ib83 and Ib85) and *Phlebiopsis gigantea* (Pg91) at two-day intervals (0-2, 2-4, etc.)

Some isolates had different growth rhythms. Growth rates measured at two-day intervals (Fig. 7) showed changes in growth of isolate Ha87 between days 6 and 12 and for Ib83 between days 4 and 8.

### ﻿Reaction types in dual cultures on MEA (Phase 2)

**Figure 8.**
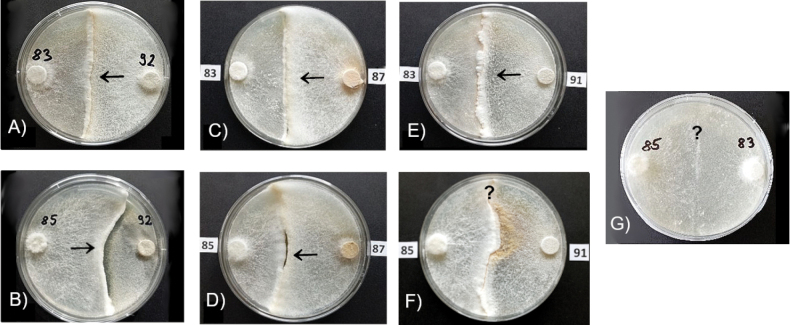
Relationships between tested isolates *Ischnoderma benzoinum* Ib83 and Ib85 with *Heterobasidion annosum* Ha92 (**A, B**), with Ha87 (**C, D**), with *Phlebiopsis gigantea* Pg91 (**E, F**), and of Ib85 with Ib83 (**G**) after 14 days in dual culture. Arrows indicate the direction of hyphae overgrowing mycelium in dual culture.

Interactions of dual cultures of isolates tested after 14 days of growth on MEA are shown in Fig. 8A–G. Isolate Ha92 showed inhibition (loser) when compared to winners Ib83 and Ib85 (Fig. 8A, B). Isolates Ib83 and Ib85 were suppressed by mycelium from both Ha87 (Fig. 8C, D) and Pa91 (Fig. 8E, F). No obvious reaction (draw) was observed in dual culture of *Ischnoderma* isolates Ib83 and Ib85 (Fig. 8G).

**Figure 9.**
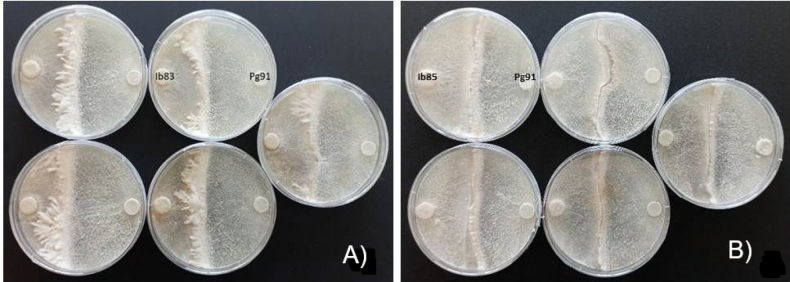
Reaction types between the mycelium of *Ischnoderma benzoinum* Ib83 (**A**) and Ib85 (**B**) and *Phlebiopsis gigantea* Pg91 in five replicates of the dual cultures on MEA after 6 weeks (Ib isolates – left in the Petri dishes, Pg isolate – right).

In tests of dual cultures on MEA carried out over 6 weeks, mycelium growth of *Ischnoderma* Ib83 was reduced when paired with isolate Pg91, based on the mutual reaction of the mycelium observed using a binocular microscope (Fig. 9A), while isolate Ib85 did not show this response (Fig. 9B).

### ﻿Wood decay in single cultures (Phase 3)

**Figure 10.**
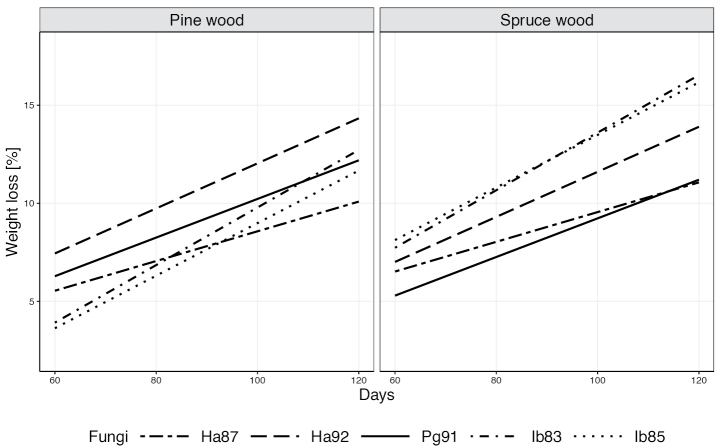
Scots pine (left) and Norway spruce (right) wood decay (weight loss %) by fungal isolates after 60 to 120 days.

The statistical summary of factors influencing wood decay is shown in Table 3 and the complicated relationships between factors influencing wood weight loss are illustrated in Fig. 10. In general, spruce samples decomposed faster than pine samples, but the hierarchy of fungal efficiency depended on wood species (shown by the statistical significance of the interaction between fungus and wood species). As expected, wood decay was greater after 120 days than after 60 days, but it should be emphasized that the hierarchy of decay efficiency changes between the 60^th^ and 120^th^ days (indicated by the statistical significance of the interaction between the fungus and number of days). The interaction is particularly pronounced for isolate Ha87, which was moderately efficient after 60 days but was least efficient in wood decay between days 60 and 120.

**Table 3.** Generalized linear model analyzing factors influencing wood decay.

Predictors	Weight loss [%]			
	Estimates	CI	Statistic	p
(Intercept)	15.99	10.34–21.63	5.55	<0.001
Ha92	-0.44	-4.28–3.39	-0.23	0.820
Pg91	-0.62	-4.48–3.23	-0.32	0.751
Ib83	-5.88	-9.70–2.07	-3.02	0.003
Ib85	-5.41	-9.22–1.59	-2.78	0.005
Spruce	0.98	-0.64–2.60	1.18	0.237
Days	0.08	0.05–0.10	5.43	<0.001
Density	-0.03	-0.04–0.02	-6.45	<0.001
Ha92:spruce	-1.41	-3.72–0.90	-1.20	0.231
Pg91:spruce	-1.97	-4.26–0.33	-1.68	0.093
Ib83:spruce	2.84	0.54–5.14	2.42	0.016
Ib85:spruce	3.52	1.22–5.81	3.00	0.003
Ha92:days	0.04	0.00–0.08	1.98	0.048
Pg91:days	0.02	-0.02–0.06	1.15	0.251
Ib83:days	0.07	0.03–0.11	3.61	<0.001
Ib85:days	0.06	0.02–0.10	2.98	0.003
Observations	200			
R2	0.727			

Comparison of the marginal mean values in Table 4 shows that after 120 days, two homogeneous groups are distinguishable for pine wood, with isolates Ha87 and Ha92 being the least and most effective decay pathogens, respectively. There are three homogeneous groups for spruce, with isolate Ha87 being the least effective agent after 120 days, whereas isolate Pg91 was the least effective after 60 days; the isolates Ib85 and Ib83 were the most effective after both periods of the wood-decay test.

**Table 4.** Decomposition (weight loss %) of pine and spruce wood after 60 and 120 days by fungal isolates and homogeneous fungal groups.

Time [days]	Isolates	Wood	Weight loss [%]	SE	Lower confidence limit	Upper confidence limit	Homogeneous group
	Ib85	pine	3.63	0.7164	2.22	5.04	A
	Ib83	pine	3.92	0.7213	2.49	5.34	A
60	Ha87	pine	5.54	0.7210	4.12	6.96	AB
	Pg91	pine	6.28	0.7167	4.87	7.70	AB
	Ha92	pine	7.44	0.7176	6.03	8.86	B
	Ha87	pine	10.08	0.7179	8.67	11.50	A
	Ib85	pine	11.67	0.7179	10.25	13.08	AB
120	Pg91	pine	12.19	0.7187	10.77	13.61	AB
	Ib83	pine	12.72	0.7242	11.29	14.15	AB
	Ha92	pine	14.33	0.7244	12.90	15.76	B
	Pg91	spruce	5.29	0.7185	3.87	6.71	A
	Ha87	spruce	6.52	0.7188	5.10	7.94	AB
60	Ha92	spruce	7.01	0.7248	5.58	8.44	AB
	Ib83	spruce	7.73	0.7175	6.32	9.15	AB
	Ib85	spruce	8.12	0.7171	6.71	9.54	B
	Ha87	spruce	11.06	0.7198	9.64	12.48	A
	Pg91	spruce	11.20	0.7168	9.78	12.61	AB
120	Ha92	spruce	13.90	0.7178	12.48	15,31	BC
	Ib85	spruce	16.16	0.7215	14.74	17.58	C
	Ib83	spruce	16.53	0.7166	15.12	17.95	C

**Figure 11.**
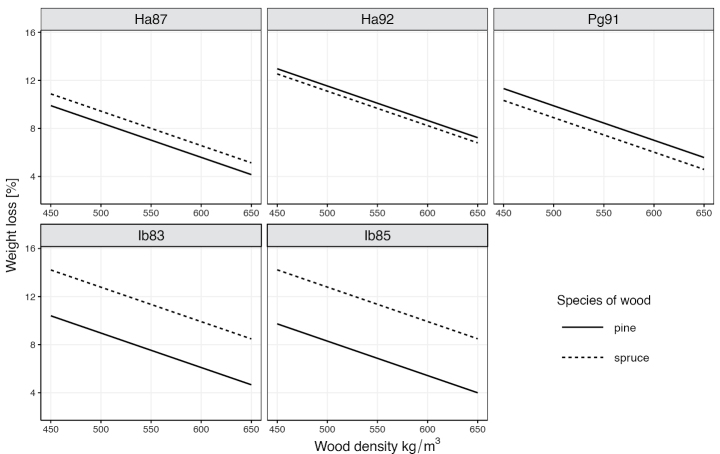
Relationships between wood density and weight loss of Scots pine and Norway spruce wood samples with different fungal isolates.

Isolates Ha92 and Pg91 decayed pine wood slightly faster than spruce wood. In contrast, the opposite situation was observed for isolates Ha87 and especially for the two *Ischnoderma* isolates, where spruce wood decay was significantly greater than that of pine wood for samples of the same wood density (Fig. 11). In both tree species, isolate Ha87 showed the lowest mass loss values after 60 and 120 days of the decay testing. Interestingly, the two *Heterobasidion* isolates showed significantly different decomposition activity of pine wood (Table 4), expressed by their belonging to different homogeneous groups. In spruce wood, on the other hand, both *Ischnoderma* isolates caused similar decomposition (same homogeneous group), but with significantly higher activity than isolate Ha87.

While the rates of wood decomposition increased over time, rates depended on wood species and fungal isolate (Fig. 10). For pine, the highest rates of decomposition were found for isolate Ha92 at both test times, whereas after 60 days decomposition was least for isolate Ib85 and after 120 days for isolate Ha87 (Table 4). Also noteworthy is the change in the decomposition rate of pine wood over time for isolate Ib83, which produced significantly higher wood weight loss from day 60 to 120 than from day 0 to 60 (Fig. 10 left). Isolate *P. gigantea* Pg91 caused mid-range pine wood decomposition compared to other isolates, while it caused the least decomposition in spruce at both days 60 and 120, with the degree of wood decomposition after 120 days almost the same for spruce as for isolate Ha87 (Fig. 10 right). In pine wood, differences in wood loss between the two *Heterobasidion* isolates were significant, while in spruce, wood loss caused by both *Ischnoderma* isolates after 60 and 120 days was higher than for other fungi.

The relationship between wood density, and thus the proportion of latewood in the annual increment of the test samples, and the degree of wood decomposition, i.e., the loss of dry wood mass, were as expected: with increasing wood density, dry matter loss decreased in both species, with values differing depending on wood species and fungal isolate (Fig. 11).

### ﻿Comparison of wood decomposition in single and dual cultures (Phases 3 and 4)

In single cultures on pine wood (Table 4, Suppl. material 1), the highest loss of dry matter of any individual wood sample was caused by isolate Ha92 (7.10%) and the lowest loss of any individual wood sample was with isolate Ib83 (3.94%). On average (i.e., for all wood samples of each isolate), the highest losses in single cultures of isolates were caused by Ib83 and Ha92 (both at 5.23%) and the lowest by Ib83 and Ha87 (both at 4.36%). In single cultures on spruce wood, the highest dry weight loss of an individual wood sample was observed for isolate Ib85 (8.23%), while the lowest individual weight loss by a wood sample was caused by isolate Ha87 (5.95%). The highest average weight loss with single-cultured isolates was caused by Ib83 and Pg91 (6.77%), the lowest by Ib83 and Ha87 (both at 4.28%).

In dual cultures, the dry weight loss of pine wood (Table 5, left part) was highest for Ib83 with Pg91 (7.50%) and lowest for Ib83 with Ha87 (3.53%), although it was not significantly different from that observed in single cultures. The high weight loss of Ib83 paired with Pg91 is noteworthy because Ib83 had one of the lower levels of wood decomposition (3.94%) when cultured alone, and Pg91 had only moderately high decomposition activity (6.01%).

**Table 5.** Average dry weight loss (%) of pine and spruce wood samples after 60 days of fungal isolate incubation in single and dual cultures.

Scots pine					Norway spruce				
Isolate confrontation		Average decay calculated from the corresponding single cultures	Average decay in dual cultures	p-value	Isolate confrontation		Average decay calculated from the corresponding single cultures	Average decay in dual cultures	p-value
Ib83	Ha92	5.53	6.13	0.5734	Ib85	Ha92	5.45	3.09	0.1608
Ib83	Ha87	4.36	3.53	0.5734	Ib85	Ha87	4.28	4.72	0.8112
Ib83	Pg91	4.95	7.50	0.1119	Ib85	Pg91	4.87	6.35	0.1608
Ib85	Pg91	4.87	5.70	0.9371	Ib83	Pg91	6.77	4.56	0.0280

Dry weight loss of spruce wood (Table 5, right part) in dual cultures was highest for the pairing of Ib85 with Pg91 (6.35%), and lowest for Ib85 with Ha92 (3.09%), although it was not significantly different from that observed in single cultures. The only significant differences in decomposition activity between single and dual cultures was observed for the pair Ib83 with Pg91.

A comparison of the wood decomposition of samples in dual cultures with the decomposition caused by single taxa (Table 5) shows that the growth of both partners on wood tends to lead to lower dry wood mass loss than in single cultures, especially in the case of spruce wood. This could indicate an inhibitory effect of the isolates, as it was found that both on MEA and in wood confrontation that there was overlap of Ib85 and Ha92 on spruce. In contrast, the presence of the competing fungus *P. gigantea* Pg91 on both pine and spruce probably indicates inhibition of the partner isolate and occupation of its food base, which is expressed by increased wood decomposition (Table 5).

The low pine wood decomposition activity in dual cultures of Ib83 and Ha87 (Table 5) could be due to the low activity of both partners, which was observed in single cultures of these isolates. In contrast, the highest weight loss of pine wood occurred in dual cultures of Ib83 with Pg91, probably due to the higher activity of the *Phlebiopsis* isolate, whose developing mycelium and secreted enzymes dominated the development of its partner, with the result of Ib85 being better on pine wood than on spruce wood.

### ﻿Reaction types in dual cultures on MEA and on wood blocks (Phases 2 and 5)

The frequency of confrontation results between selected isolates on MEA and wood is shown in Table 6. The outcomes were statistically analyzed in two ways. First, independence between the result of confrontation and the identity of the competitor was tested separately for both substrates for each *Ischnoderma* isolate. The resulting p-values are not given in Table 6 but are presented in text. Fisher’s exact test confirmed that the confrontation results for Ib83 on both MEA (p-value = 0.034) and wood (p-value = 0.004) were dependent on the identity of the competing isolate. For Ib85, confrontation results were also dependent on the identity of the competing isolate on MEA (p-value = 0.015) and wood (p-value = 0.026).

**Table 6.** Results of confrontations between mycelium of isolates of *Ischnoderma benzoinum* (Ib83 and Ib85) versus *I. benzoinum*, *H. annosum* and *P. gigantea* on malt extract agar (MEA) in Phase 2 and on pine and spruce wood in Phase 5. Results indicating Loser, Draw and Winner and p-values represent the outcome of the Fisher test based on five replicate Petri dishes (MEA) and six wood samples (wood) concerning independence between substrate type and the outcome of confrontation.

Isolate	Substrate	p-value	Ib83			p-value	Ib85		
			Loser	Draw	Winner		Loser	Draw	Winner
**Ib83**	MEA		not tested			0.015	1	2	2
	wood						–	–	6
**Ib85**	MEA	0.015	2	2	1		not tested		
	wood		6	–	–				
**Ha87**	MEA	0.455	5	–	–	0.999	5	–	–
	wood		4	–	2		5	–	1
**Ha92**	MEA	0.546	1	1	3	0.455	–	–	5
	wood		3	–	3		2	–	4
**Pg91**	MEA	0.999	5	–	–	0.455	4	1	–
	wood		6	–	–		6	–	–

Secondly, based on results in Table 6, independence of the confrontation results from substrate type was also tested for each isolate pair presented (e.g. Ha87 vs. Ib85). Only for isolates Ib83 and Ib85 was the result of the confrontation influenced by substrate (p-value = 0.015).

Comparison of raw confrontation results provides additional interesting insights. In the direct confrontation of *Ischnoderma* isolates with the pathogen *Heterobasidion*, we noted that mycelium of isolate Ib85 (from pine) strongly reduced the growth of isolate Ha92 (from pine) but was the loser in the confrontation with isolate Ha87 (from spruce). In contrast, the mycelium of isolate Ib83 (from spruce) was reduced by the mycelium of Ha87 (from spruce).

The interaction of *Ischnoderma* Ib85 with *H. annosum* strongly differed depending on the *Heterobasidion* isolate, with Ha87 clearly dominant over Ib85, especially on wood samples. In contrast, Ib85 strongly reduced mycelial growth of the pathogen Ha92. Substrate (agar or wood) did not significantly influence the outcome of the confrontation between Ib85 and Ha87. *Ischnoderma* isolates Ib83 and Ib85, the substrate influenced the outcome of the interaction of these isolates (p-value = 0.01515).

**Figure 12.**
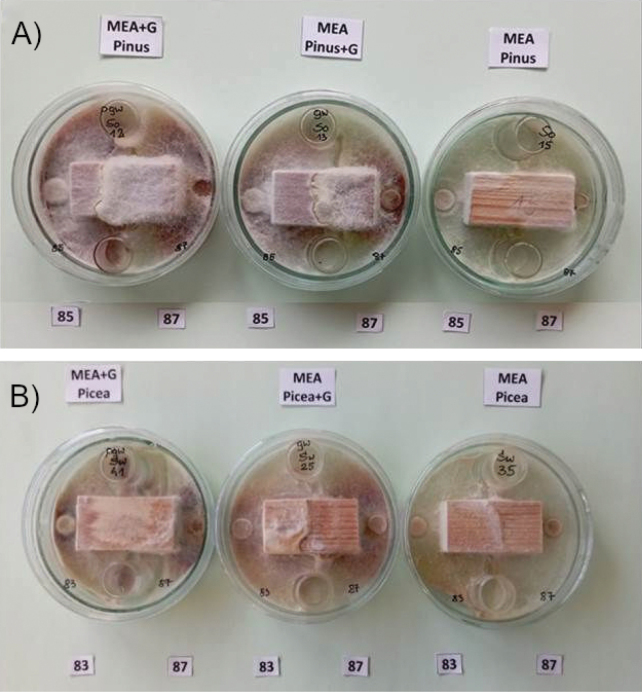
Examples of reaction types of mycelia decomposing pine wood by isolates *Ischnoderma benzoinum* Ib85 and *Heterobasidion annosum* Ha87 (**A**) and decomposing spruce wood by Ib83 and Ha87 (**B**) on MEA+G (left), MEA and wood saturated with guaiacol (centre) and MEA (right) after 60 days incubation.

The effect of the dominance of mycelium of Ha87 (winner) over mycelium of Ib85 (loser) is evident in pine wood samples with guaiacol (+G) substrates – the color reaction facilitated the determination of the reaction zones and the direction of mycelium growth (Fig. 12).

**Figure 13.**
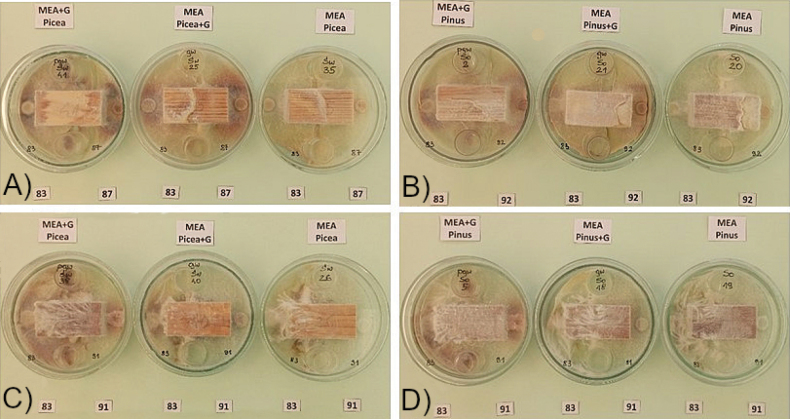
Reaction types between aerial mycelium of isolate *Ischnoderma benzoinum* Ib83 and *Heterobasidion annosum* on spruce wood Ha87 (**A**), and Ib83 and *H. annosum* Ha92 (**B**) on pine wood, and Ib83 and *Phlebiopsis gigantea* Pg91 on spruce wood (**C**) and Ib83 and Pg91 on pine wood (**D**) in Petri dishes containing MEA with guaiacol (left), MEA and wood saturated with guaiacol (middle), or MEA (right) after six weeks in Petri dishes.

Interactions of the five isolates tested in pairs (see Table 5, 6) for 3 replicates were more pronounced on pine wood than on spruce. Differences also depended on the type of wood samples, especially in samples saturated with guaiacol (Fig. 13). It is interesting to note the opposite effect of isolate Ha87 and the two isolates Ib85 and Ib83 on spruce wood for all three of these substrates, which could indicate interactions of oxidative enzymes of the two fungi, expressed by the intensity of the reaction in the presence of guaiacol. The comparison of mycelial development and color symptoms (Doerge et al. 1997; Tsigain et al. 2022) on rotting wood blocks by *Ischnoderma* isolate Ib83 (from spruce) confronted in dual cultures with Ha87 (from spruce) (Fig. 12B) or with Ha92 (from pine) shows the different characteristics of the interactions. While confrontation with Ha87 shows clear pathogen dominance, confrontation with Ha92 shows an unclear state of equilibrium, indicating a competitive effect of this *Ischnoderma* isolate against the pathogen.

**Figure 14.**
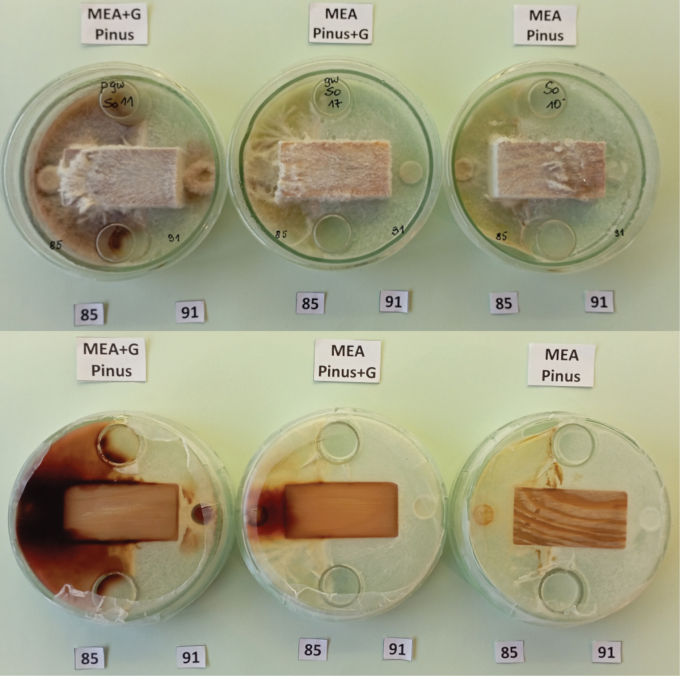
Front (upper) and backside (lower) of dishes with pine samples covered with Ib85 and Pg91 isolates on MEA+G medium with guaiacol (left), wood saturated with guaiacol (center), and on MEA medium (right).

The intense color changes of isolates Ha87 (Fig. 13A) and Ha92 (Fig. 13B) in media with guaiacol (+G) confirm the pathogenic ability of these *Heterobasidion* isolates. On the other hand, the lack of a color reaction of Pg91 in both +G guaiacol variants (Fig. 13C, D) and a clear mycelial overgrowth of the two wood block species over mycelium from Ib83 shows the competitive ability of Pg91 against this isolate. Based purely on an in vitro confrontation test, the above observations indicate that the pine-derived *P. gigantea* strain can restrict the growth of Ib83 on both pine and spruce substrates (Fig. 13), albeit to a lesser extent on spruce. The color change of MEA with guaiacol can be better observed on the back of the Petri dishes due to the enzymatic production of the growing mycelium of the isolate Ib85 (Fig. 14).

**Figure 15.**
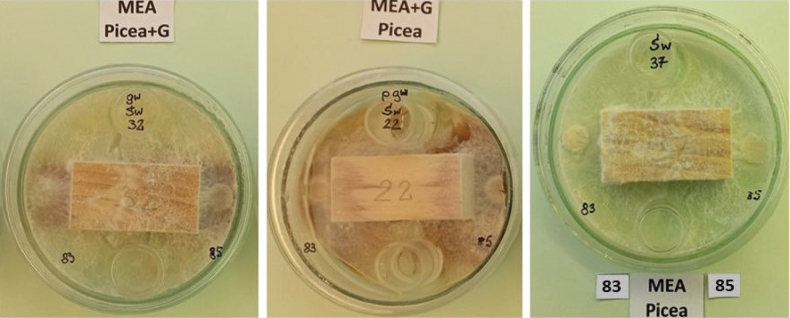
Absence of a line of demarcation between the mycelium of two *Ischnoderma* isolates (Ib85 and Ib83) on MEA, with or without guaiacol in spruce wood after 14 days of growth.

The growth of the *Ischnoderma* isolates on spruce samples (Fig. 15) showed production of oxidase enzymes on the medium (brown color) and weak development of aerial mycelium, without a demarcation zone between the two cultures, similar to dual cultures on MEA.

## ﻿Discussion

Wood decay depends on enzymatic decomposition of the cell wall lignin-cellulose complex. White-rot fungi target lignin degradation of latewood, and cellulose is degraded by hydrolytic cellulases and subsequently produced dehydrogenases and oxidases (Henriksson et al. 1995; Reddy and D’Souza 1998; Sigoillot et al. 2005; Sierota et al. 2015). Enzyme production depends on the source of the mycelium and the genetic characteristics of the isolates and is higher in wood mycelium than in aerial mycelium during wood decomposition, which does not always correspond to the growth of the isolates on media (Żółciak et al. 2012).

We are unaware of studies evaluating pathogenicity of *I. benzoinum*. According to Rayner and Boddy (1988), this fungus can be an active decomposer or a latent invader in a standing tree before it falls. This is confirmed by our observations of some dead pines and spruces with *I. benzoinum* basidiomata growing at the base of the trunks. *Ischnoderma benzoinum* basidiomata on dead wood (stumps and trunks) can be confused with *Heterobasidion* spp. due to the great similarity of its hymenophor and upper surface, which may affect the reliability of fungal hazard assessments. According to Niemelä and Kotiranta (1986), *I. benzoinum* could be a weak parasite, but there is no direct evidence supporting this claim. The authors cited above believe that economic losses to *I. benzoinum* are low, as the rot progresses very slowly so that other fungi already infest logs before it progresses very far.

According to Stalpers (1978), the growth radius of *I. benzoinum* colonies on MEA medium is 45–70 mm after two weeks. This growth rate is similar to that of *H. annosum* (35–70 mm), but some isolates of *H. annosum* rarely reach 15–35 mm (Stalpers 1978). Napierała-Filipiak and Werner (2000) noted rapid growth (colony diameter 7.34–9.24 cm after two weeks) of three isolates of *H. annosum* on pb basic medium. In general, cultures of the competitor *P. gigantea* grow more actively than *Ischnoderma* and *Heterobasidion*. For example, three Latvian strains of *P. gigantea* are characterized by a growth rate of 6.5 to 8.0 mm per day (Brûna et al. 2020). Sun et al. (2009) found that the radial growth of *P. gigantea* colonies in agar ranges from 8–10.8 mm per day, similar to Żółciak et al. (2016), who describe the growth rate of this fungus at up to 20–23 mm/day, while similar *H. parviporum* grows 16–17 mm per day, but as colony diameter (Żółciak et al. 2016). Under conditions in the present study, the growth of the *P. gigantea* isolate on MEA was the slowest among all isolates.

It is noteworthy that *Ischnoderma* isolates tested on MEA in Petri dishes did not form a demarcation line or a denser layer of hyphae at the point of direct contact in dual culture, although demarcation was observed in other variants. Our study shows that both isolates of *I. benzoinum* caused substantial mass loss of pine and spruce wood under laboratory conditions. Among the fungal species tested, both *Ischnoderma* isolates (Ib83 and Ib85) resulted in the least decomposition of pine wood after 60 days of the wood-decay test. After 120 days, the pine isolate Ib85 continued to show some of the lowest levels of decomposition activity. The spruce isolate of *Ischnoderma* (Ib83) caused a slightly greater mass loss of pine wood after 120 days than the pine isolate (Ib85). In contrast, both *Ischnoderma* isolates (Ib83 and Ib85) caused the greatest mass loss of spruce wood after 60 and 120 days. However, after 60 days, the *Ischnoderma* isolate from pine (Ib85) decomposed spruce wood slightly more than the isolate from spruce (Ib83). After 120 days, this trend reversed, with the spruce isolate (Ib83) having only somewhat greater effect. Fungi that cause white rot on wood mainly degrade lignin and hemicellulose (Blanchette 1984; Leviu and Castro 1998; Schwarze et al. 2000; Wang et al. 2013; Daniel 2016; Li et al. 2025), and have little effect on cellulose, depending on culture conditions. However, Sutherland (1986) showed that *I. resinosum*, which is morphologically similar to *I. benzoinum*, can inhibit cellulase activity in the presence of xylose and other sugars found in wood hemicellulose. Structural differences in the cell wall structure of pine and spruce wood, e.g., wood density and lignin and hemicellulose content, may also influence decomposition by the isolates studied, and must be taken into account (Alves et al. 2006; Sierota et al. 2016; Żółciak et al. 2016).

*Heterobasidion* generally exhibits strong decomposition properties (Maijala 2000). For example, decomposition (dry weight loss) of Scots pine wood by *H. annosum* was 3.4% after two months and by *H. parviporum* 2.8% (Negrutsky et al. 1994 after Woodward et al. 1998). Weight loss of Norway spruce wood due to *H. parviporum* was 18.9% after 3 months (Żółciak et al. 2016). Worrall et al. (1997) reported wood mass losses by *H. annosum* in a 12-week soil block rot experiment of yellow birch (*Betula alleghaniensis* Britton) of 2.2% and loblolly pine (*Pinus taeda* L.) of 8.9%. Weight loss of beech wood (*Fagus sylvatica* L.) after 4 months due to *H. annosum* was 5–6% (Schmidt et al. 1986). Szczepkowski et al. (2025) found wood mass losses by *H. annosum* in a 4-month experiment of black locust (*Robinia pseudoacacia* L.) ranged 0–1.24%, whereas in beech it ranged 10.62–15.34%. In the present experiment, weight loss after a 120-day pine wood decay test with *P. gigantea* isolate Pg91, the comparative isolate of the saprotrophic fungus, averaged 12.19%, similar to both *Ischnoderma* isolates (Ib83–12.73%, Ib85–11,67%) but lower than for isolate Ha92 from pine (14.33%). In the case of spruce wood, the lowest decay levels after 120 days were by *Heterobasidion* isolate Ha87 obtained from spruce (11.06%) and by isolate Pg91 (11.20%).

Rykowski and Sierota (1977), Żółciak et al. (2008, 2012), and Cartabia et al. (2021) observed that mycelial migration through various natural substrates, including wood as a source of nutrients, promotes hyphal growth and wood decomposition activity of many fungal species originating from “maternal” bases (basidiomata, wood of the other species). The root rot fungus *Armillaria ostoyae* (Romagn.) Herink is particularly active against pine roots when its infectious rhizomorphs feed on oak or hazelwood (Kwaśna et al. 2001; Heinzelmann et al. 2017). Pratt et al. (2000) described differences in mycelial activity associated with commercial products of *P. gigantea*, which can be attributed to the source of the maternal mycelium, among other factors. Rykowski and Sierota (1977) found that the growth of *P. gigantea* mycelium on pine stumps was best when the inoculum passed through beech sawdust, although passage of the mycelium probably reduces its activity over time (D’Souza 1999).

Adding guaiacol to growth medium or wood allowed the presence and range of action of the phenoloxidase enzyme produced by fungal isolates to be identified. Dark brown coloration was the result of guaiacol oxidation by phenoloxidase, as described by Boidin (1951) and Havličková and Rypáček (1957). Specific interactions, such as hyphae overgrowing the mycelium of the co-partner, a dense layer of hyphae, or an inhibition zone, were related to increased enzyme production and reduced wood decomposition observed on both the front and back of some plates in dual cultures, which was also observed by Molla et al. (2001) in their research on bioconversion processes. In our research, this reaction was observed for both *Heterobasidion* isolates and to a lesser extent for both *Ischnoderma* isolates. In contrast, *P. gigantea* isolate Pg91 did not stain medium containing guaiacol, confirming that this strain is saprotrophic and does not produce phenoloxidase (Sierota et al. 2015; Żółciak et al. 2020).

Many fungal species have been tested in vitro and in vivo in the search for natural antagonists against *H. annosum* (Woodward et al. 1998). Rishbeth (1970), who evaluated antagonism of *I. benzoinum* against *H. annosum* on inoculated stumps of *Picea abies* and *P. sitchensis* (Bong.) Carrière, among others, described the result as “weak or no antagonistic activity.” A similar result was found by Hanso (1993), as reported by Woodward et al. (1998), when comparing the growth of dual cultures of *I. benzoinum* and *H. annosum* on malt agar. Watanabe et al. (2003) and Nawrot-Chorabik et al. (2024) also described competition between different wood-decaying species cultured in pairs. Our studies revealed isolate dependence on substrate in determining winners and losers in dual cultures on MEA and wood blocks. Similarly, Tanaka et al. (2000) indicated that fungal phenoloxidase is active in wood cultures but not in glucose-rich cultures. And Giles et al. (2015) found that competition in wood decomposition in dual cultures of white and brown rot taxa reduced mass loss and some wood chemicals (e.g. hemicelluloses), but had no effect on sugar yield compared to single cultures, suggesting a limited stimulatory or an inhibitory effect of joint growth. When we compared white-rot fungi in dual cultures, higher wood loss in dual culture versus culture of fungi separately was observed only for Pg91 from pine and Ib83 from spruce when growing on pine wood. In other cases, wood loss was lower in dual cultures, which could indicate enzymatic competition between the paired taxa and inhibition of wood decomposition. The case of Pg91 and Ib83 shows competition between these taxa and accelerated wood decomposition by the *P. gigantea* strain. In our experiments, the responses of *I. benzoinum* to *H. annosum* were not as clear as those described by Otto and Blanchette (2023) in studies on *Heterobasidion irregulare* Garbel.

Spruce wood was decomposed differently than pine wood by the various isolates. Isolate Ib83, which was only weakly active on pine wood, proved to be more active on spruce, as was isolate Ib85. This could indicate that compounds in the cell walls of spruce wood more strongly promote mycelial growth and catalyze decomposition reactions. A similar response to aqueous pine bark extracts on wood-decaying fungi is described by Morita et al. (2001). Both *Heterobasidion* isolates showed similar wood decomposition activity in pine and spruce, as did *P. gigantea* Pg91. In the case of *Ischnoderma* and *Heterobasidion* species in dual cultures, wood decomposition results should account for both the activity of aerial mycelium in cultures on agar and degree of development on wood blocks, which is facilitated by the color change of guaiacol. Based on these observations, it can be seen that both *Ischnoderma* isolates strongly dominate the development of *Heterobasidion* Ha92 (isolated from pine wood) – in cultures on agar and on wood. At the same time, *Ischnoderma* isolates on both agar and on wood were inferior to Ha87 (isolated from spruce wood). And *Ischnoderma* and *Heterobasidion* were both dominated by isolate Pg91 from *Phlebiopsis gigantea* on agar and on wood.

In this experiment we compared mycelium of three fungal species on the same substrates (MEA and wood) that potentially occupy the same food bases – pine and spruce wood. We found considerable competitiveness between some isolates, even within the same fungal species. Another aspect of such a balanced effect was the source of the isolates – whether from pine or spruce food bases. The level of decay among single and dual cultures also depended on the food base from which the isolate was obtained. Furthermore, in some cases, dual cultures resulted in higher decay rates than single isolates, contradicting the claim that dual cultures generally reduce mycelial growth or wood decomposition (Giles et al. 2015; Cui et al. 2023; Nawrot-Chorabik et al. 2024).

These observations, reinforced by the results of the color reactions with guaiacol in the laboratory, confirm that the *Ischnoderma* isolates studied may have a similar pathogenicity and wood decomposing ability as the *Heterobasidion* isolates. It isn’t easy to give a definite answer to the essence of *Ischnoderma*’s functioning, just as it is challenging to transfer laboratory results to a forest ecosystem. Based on our results, the answer to the question raised in this study, – whether *Ischnoderma* is a potential pathogen or competitor of *Heterobasidion* or only contributes to the decomposition of pine or spruce wood, depends on the presence of germinating spores and then mycelium and basidiomata on pine or spruce wood in nature as well as on the individual resistance of the trees. This question requires a more detailed genetic study. We are aware of some limitations of the study, such as the host source, the number of isolates (which is due to the low availability of *Ischnoderma* basidiomata in commercial stands), and the limited number of samples used to assess the degree of wood decay in dual culture and the interaction between the two cultures. We hope for an improvement in future studies.

## ﻿Conclusions

*Ischnoderma* had high levels on mycelial growth of MEA medium and very active decomposition of pine and spruce wood, indicating that this fungus can participate in the decomposition of root and stump wood in stands.

The reaction of *Ischnoderma* confronted with the pathogen *Heterobasidion* depends on the mycelial activity of specific isolates and the activity of the pathogen isolate, both of which depend on the type of infested wood (spruce or pine) and the host of isolate originating from pine or spruce substrates.

The simultaneous presence of two *Ischnoderma* isolates has no significant influence on the degree of decomposition of the two wood species.

Of the two *Ischnoderma* isolates used in the study, the isolate originating from pine was less active than that from spruce.

Two *Ischnoderma* isolates and a *P. gigantea* isolate show similar competitive abilities to grow and decompose wood only versus the spruce-derived *Heterobasidion* isolate Ha87.

*Phlebiopsis gigantea* was highly effective in limiting mycelial growth of the two *Ischnoderma* taxa and in decomposing wood colonized by these species.

*Ischnoderma* basidiomata can be confused with *Heterobasidion*, affecting the reliability of disease risk assessment in managed forests.

## ﻿Additional information

### Use of AI

No use of AI was reported.

### Adherence to national and international regulations

All the fungal strains used in this study have been legally obtained, respecting the Convention on Biological Diversity (Rio Convention).

### Author ORCIDs

Andrzej Szczepkowski https://orcid.org/0000-0002-9778-9567

Leszek Bolibok https://orcid.org/0000-0003-4395-435X

Zbigniew Sierota https://orcid.org/0000-0003-3419-7799

### Data availability

All of the data that support the findings of this study are available in the main text or Supplementary Information.

## Supplementary materials

Supplementary material 1 Raw measurement data from phases 1 and 3 of the experiment Data type docx Explanation note The first phase of the experiment: evaluation of the linear growth of five isolates of the fungi tested on MEAThe third phase of the experiment: evaluation of the extent of wood dry matter loss in Scots pine and spruce samples separately caused by isolates tested on MEA. https://binary.pensoft.net/file/1412007This dataset is made available under the Open Database License (http://opendatacommons.org/licenses/odbl/1.0/). The Open Database License (ODbL) is a license agreement intended to allow users to freely share, modify, and use this Dataset while maintaining this same freedom for others, provided that the original source and author(s) are credited.Andrzej Szczepkowski, Leszek Bolibok, Zbigniew Sierota
