# Supplementary material for: ﻿Is Ischnoderma benzoinum a competitor or contributor to Heterobasidion annosum decomposition of pine and spruce wood? A comparison to Phlebiopsis gigantea
Source: IMA Fungus. 2025 Sep 9;16:e152556. doi: 10.3897/imafungus.16.152556 (PMC12441746; doi:10.3897/imafungus.16.152556)
Supplement: Supplementary material 1 — Raw measurement data from phases 1 and 3 of the experiment [file imafungus-16-e152556-s001.docx]

The first phase of the experiment: evaluation of the linear growth of five isolates of the fungi tested on MEA

No. Isolate No.2 Day Range

1 Ha87 1 2 4.0

2 Ha87 2 2 5.0

3 Ha87 3 2 5.0

4 Ha87 4 2 2.0

5 Ha87 5 2 2.0

6 Ib83 1 2 2.0

7 Ib83 2 2 1.0

8 Ib83 3 2 1.0

9 Ib83 4 2 1.0

10 Ib83 5 2 2.0

11 Ib85 1 2 1.0

12 Ib85 2 2 2.0

13 Ib85 3 2 2.0

14 Ib85 4 2 1.0

15 Ib85 5 2 1.0

16 Pg91 1 2 5.0

17 Pg91 2 2 5.0

18 Pg91 3 2 6.0

19 Pg91 4 2 3.0

20 Pg91 5 2 2.0

21 Ha92 1 2 3.0

22 Ha92 2 2 3.0

23 Ha92 3 2 2.0

24 Ha92 4 2 3.0

25 Ha92 5 2 3.0

26 Ha87 1 4 23.0

27 Ha87 2 4 20.0

28 Ha87 3 4 22.0

29 Ha87 4 4 11.0

30 Ha87 5 4 12.0

31 Ib83 1 4 18.0

32 Ib83 2 4 11.0

33 Ib83 3 4 7.0

34 Ib83 4 4 12.0

35 Ib83 5 4 19.0

36 Ib85 1 4 14.0

37 Ib85 2 4 13.0

38 Ib85 3 4 12.0

39 Ib85 4 4 8.0

40 Ib85 5 4 7.0

41 Pg91 1 4 21.0

42 Pg91 2 4 20.0

43 Pg91 3 4 22.0

44 Pg91 4 4 15.0

45 Pg91 5 4 12.0

46 Ha92 1 4 18.0

47 Ha92 2 4 15.0

48 Ha92 3 4 18.0

49 Ha92 4 4 21.0

50 Ha92 5 4 14.0

51 Ha87 1 6 39.0

52 Ha87 2 6 36.0

53 Ha87 3 6 38.0

54 Ha87 4 6 2.5

55 Ha87 5 6 27.0

56 Ib83 1 6 28.0

57 Ib83 2 6 20.0

58 Ib83 3 6 20.0

59 Ib83 4 6 25.0

60 Ib83 5 6 28.0

61 Ib85 1 6 29.0

62 Ib85 2 6 28.0

63 Ib85 3 6 27.0

64 Ib85 4 6 23.0

65 Ib85 5 6 22.0

66 Pg91 1 6 34.0

67 Pg91 2 6 34.0

68 Pg91 3 6 39.0

69 Pg91 4 6 30.0

70 Pg91 5 6 25.0

71 Ha92 1 6 35.0

72 Ha92 2 6 33.0

73 Ha92 3 6 28.0

74 Ha92 4 6 38.0

75 Ha92 5 6 30.0

76 Ha87 1 8 54.0

77 Ha87 2 8 50.0

78 Ha87 3 8 53.0

79 Ha87 4 8 38.0

80 Ha87 5 8 41.0

81 Ib83 1 8 45.0

82 Ib83 2 8 38.0

83 Ib83 3 8 37.0

84 Ib83 4 8 42.0

85 Ib83 5 8 44.0

86 Ib85 1 8 46.0

87 Ib85 2 8 45.0

88 Ib85 3 8 42.0

89 Ib85 4 8 39.0

90 Ib85 5 8 38.0

91 Pg91 1 8 50.0

92 Pg91 2 8 48.0

93 Pg91 3 8 53.0

94 Pg91 4 8 43.0

95 Pg91 5 8 40.0

96 Ha92 1 8 54.0

97 Ha92 2 8 53.0

98 Ha92 3 8 48.0

99 Ha92 4 8 57.0

100 Ha92 5 8 48.0

101 Ha87 1 10 73.0

102 Ha87 2 10 69.0

103 Ha87 3 10 72.0

104 Ha87 4 10 57.0

105 Ha87 5 10 60.0

106 Ib83 1 10 62.0

107 Ib83 2 10 55.0

108 Ib83 3 10 55.0

109 Ib83 4 10 60.0

110 Ib83 5 10 61.0

111 Ib85 1 10 63.0

112 Ib85 2 10 62.0

113 Ib85 3 10 58.0

114 Ib85 4 10 57.0

115 Ib85 5 10 57.0

116 Pg91 1 10 66.0

117 Pg91 2 10 63.0

118 Pg91 3 10 69.0

119 Pg91 4 10 59.0

120 Pg91 5 10 51.0

121 Ha92 1 10 73.0

122 Ha92 2 10 73.0

123 Ha92 3 10 67.0

124 Ha92 4 10 77.0

125 Ha92 5 10 68.0

126 Ha87 1 12 93.0

127 Ha87 2 12 86.0

128 Ha87 3 12 90.0

129 Ha87 4 12 73.0

130 Ha87 5 12 78.0

131 Ib83 1 12 80.0

132 Ib83 2 12 72.0

133 Ib83 3 12 72.0

134 Ib83 4 12 77.0

135 Ib83 5 12 77.0

136 Ib85 1 12 81.0

137 Ib85 2 12 80.0

138 Ib85 3 12 76.0

139 Ib85 4 12 75.0

140 Ib85 5 12 75.0

141 Pg91 1 12 82.0

142 Pg91 2 12 80.0

143 Pg91 3 12 86.0

144 Pg91 4 12 75.0

145 Pg91 5 12 66.0

146 Ha92 1 12 94.0

147 Ha92 2 12 94.0

148 Ha92 3 12 87.0

149 Ha92 4 12 97.0

150 Ha92 5 12 87.0

151 Ha87 1 14 113.0

152 Ha87 2 14 104.0

153 Ha87 3 14 110.0

154 Ha87 4 14 93.0

155 Ha87 5 14 95.0

156 Ib83 1 14 98.0

157 Ib83 2 14 90.0

158 Ib83 3 14 87.0

159 Ib83 4 14 97.0

160 Ib83 5 14 95.0

161 Ib85 1 14 100.0

162 Ib85 2 14 98.0

163 Ib85 3 14 95.0

164 Ib85 4 14 94.0

165 Ib85 5 14 95.0

166 Pg91 1 14 100.0

167 Pg91 2 14 96.0

168 Pg91 3 14 100.0

169 Pg91 4 14 92.0

170 Pg91 5 14 80.0

171 Ha92 1 14 116.0

172 Ha92 2 14 115.0

173 Ha92 3 14 108.0

174 Ha92 4 14 116.0

175 Ha92 5 14 108.0

176 Ha87 1 16 120.0

177 Ha87 2 16 120.0

178 Ha87 3 16 120.0

179 Ha87 4 16 108.0

180 Ha87 5 16 115.0

181 Ib83 1 16 115.0

182 Ib83 2 16 105.0

183 Ib83 3 16 105.0

184 Ib83 4 16 113.0

185 Ib83 5 16 110.0

186 Ib85 1 16 116.0

187 Ib85 2 16 115.0

188 Ib85 3 16 110.0

189 Ib85 4 16 110.0

190 Ib85 5 16 112.0

191 Pg91 1 16 113.0

192 Pg91 2 16 110.0

193 Pg91 3 16 112.0

194 Pg91 4 16 105.0

195 Pg91 5 16 94.0

196 Ha92 1 16 120.0

197 Ha92 2 16 120.0

198 Ha92 3 16 120.0

199 Ha92 4 16 120.0

200 Ha92 5 16 120.0

The third phase of the experiment:
evaluation of the extent of wood dry matter loss
in Scots pine and spruce samples separately
caused by isolates tested on MEA

| No.1 | No.2 | Wood | Day | Isolate | Source | Density | Moisture after [percent] | Dry start weight | Dry end weight | Weight loss [percent] |
| --- | --- | --- | --- | --- | --- | --- | --- | --- | --- | --- |

1 1 pine 60 Ib85 Pinus 495.30 138.16 8.18 7.510 8.21

2 2 pine 60 Ib85 Pinus 481.21 126.94 8.25 8.040 2.55

3 3 pine 60 Ib85 Pinus 500.78 172.47 8.63 8.230 4.68

4 4 pine 60 Ib85 Pinus 581.57 133.73 10.01 9.720 2.95

5 5 pine 60 Ib85 Pinus 501.57 167.70 8.61 8.440 1.97

6 6 pine 60 Ib85 Pinus 474.90 172.25 8.10 7.760 4.18

7 7 pine 60 Ib85 Pinus 577.53 136.18 9.47 9.300 1.80

8 8 pine 60 Ib85 Pinus 483.21 121.03 8.24 7.830 4.92

9 9 pine 60 Ib85 Pinus 629.24 75.73 10.36 10.140 2.12

10 10 pine 60 Ib85 Pinus 571.03 134.74 9.77 9.470 2.99

11 1 pine 60 Ib83 Picea 500.27 102.52 8.38 8.140 2.90

12 2 pine 60 Ib83 Picea 473.24 134.46 7.98 7.850 1.60

13 3 pine 60 Ib83 Picea 455.72 151.52 7.67 6.920 9.78

14 4 pine 60 Ib83 Picea 575.46 138.51 9.92 9.570 3.52

15 5 pine 60 Ib83 Picea 649.61 65.09 10.85 10.500 3.23

16 6 pine 60 Ib83 Picea 632.54 62.14 10.51 10.160 3.30

17 7 pine 60 Ib83 Picea 567.56 145.16 9.63 9.310 3.32

18 8 pine 60 Ib83 Picea 493.08 169.52 8.22 8.010 2.54

19 9 pine 60 Ib83 Picea 459.06 181.77 7.43 7.000 5.90

20 10 pine 60 Ib83 Picea 566.00 133.58 9.63 9.450 1.83

21 1 pine 60 Ha92 Pinus 570.30 148.49 9.38 8.900 5.17

22 2 pine 60 Ha92 Pinus 565.81 81.79 9.68 8.580 11.31

23 3 pine 60 Ha92 Pinus 481.60 135.33 8.17 7.660 6.17

24 4 pine 60 Ha92 Pinus 612.77 97.78 10.12 9.900 2.19

25 5 pine 60 Ha92 Pinus 490.52 89.73 8.34 7.860 5.70

26 6 pine 60 Ha92 Pinus 464.50 153.10 7.73 6.690 13.56

27 7 pine 60 Ha92 Pinus 621.71 53.10 10.28 9.620 6.42

28 8 pine 60 Ha92 Pinus 622.08 47.76 10.24 9.770 4.58

29 9 pine 60 Ha92 Pinus 459.05 197.48 7.83 6.950 11.27

30 10 pine 60 Ha92 Pinus 483.33 77.95 8.14 7.630 6.23

31 1 pine 60 Ha87 Picea 584.02 119.52 9.97 9.400 5.64

32 2 pine 60 Ha87 Picea 575.12 45.58 9.43 9.350 0.85

33 3 pine 60 Ha87 Picea 473.56 115.70 8.01 7.300 8.84

34 4 pine 60 Ha87 Picea 490.86 150.20 8.46 7.780 7.99

35 5 pine 60 Ha87 Picea 571.68 139.09 9.54 9.360 1.88

36 6 pine 60 Ha87 Picea 498.36 162.33 8.52 8.290 2.76

37 7 pine 60 Ha87 Picea 575.75 112.33 9.78 9.110 6.91

38 8 pine 60 Ha87 Picea 502.23 85.38 8.43 8.210 2.66

39 9 pine 60 Ha87 Picea 573.81 109.72 9.84 9.190 6.67

40 10 pine 60 Ha87 Picea 613.74 115.82 10.73 10.190 4.99

41 1 pine 60 Pg91 Pinus 503.47 162.27 8.43 8.210 2.64

42 2 pine 60 Pg91 Pinus 479.12 111.56 8.08 7.440 7.90

43 3 pine 60 Pg91 Pinus 457.93 120.80 7.80 7.100 8.98

44 4 pine 60 Pg91 Pinus 498.53 100.24 8.43 8.200 2.73

45 5 pine 60 Pg91 Pinus 486.55 144.72 8.25 7.420 10.09

46 6 pine 60 Pg91 Pinus 585.83 140.25 10.16 9.950 2.01

47 7 pine 60 Pg91 Pinus 571.04 101.02 9.63 8.920 7.40

48 8 pine 60 Pg91 Pinus 566.31 121.69 9.61 8.940 7.01

49 9 pine 60 Pg91 Pinus 457.20 145.42 7.47 6.900 7.60

50 10 pine 60 Pg91 Pinus 455.65 170.74 7.85 7.480 4.76

51 1 pine 120 Ib85 Pinus 467.19 133.01 7.79 6.440 17.33

52 2 pine 120 Ib85 Pinus 477.28 141.21 8.14 7.250 10.96

53 3 pine 120 Ib85 Pinus 576.31 141.10 9.77 8.970 8.20

54 4 pine 120 Ib85 Pinus 494.19 170.83 8.47 7.590 10.34

55 5 pine 120 Ib85 Pinus 474.93 125.34 8.11 7.230 10.93

56 6 pine 120 Ib85 Pinus 629.37 126.19 10.39 9.550 8.14

57 7 pine 120 Ib85 Pinus 485.80 126.11 8.37 7.280 13.09

58 8 pine 120 Ib85 Pinus 476.02 153.03 8.00 6.170 22.89

59 9 pine 120 Ib85 Pinus 499.36 160.35 8.35 7.770 6.97

60 10 pine 120 Ib85 Pinus 487.44 162.00 8.19 7.350 10.28

61 1 pine 120 Ib83 Picea 573.88 111.19 9.59 8.760 8.64

62 2 pine 120 Ib83 Picea 576.10 140.39 9.95 8.910 10.43

63 3 pine 120 Ib83 Picea 570.40 141.45 9.70 8.880 8.47

64 4 pine 120 Ib83 Picea 495.01 174.84 8.44 7.530 10.76

65 5 pine 120 Ib83 Picea 573.66 139.38 9.70 8.700 10.31

66 6 pine 120 Ib83 Picea 629.42 70.71 10.46 9.790 6.38

67 7 pine 120 Ib83 Picea 561.68 137.30 9.73 8.410 13.53

68 8 pine 120 Ib83 Picea 469.71 157.84 7.92 6.050 23.66

69 9 pine 120 Ib83 Picea 574.18 131.77 9.58 8.440 11.84

70 10 pine 120 Ib83 Picea 483.08 145.00 8.09 7.110 12.14

71 1 pine 120 Ha92 Pinus 627.06 59.65 10.43 8.990 13.80

72 2 pine 120 Ha92 Pinus 561.41 121.65 9.43 8.160 13.47

73 3 pine 120 Ha92 Pinus 501.06 137.89 8.34 6.940 16.77

74 4 pine 120 Ha92 Pinus 466.64 150.92 7.90 6.400 18.99

75 5 pine 120 Ha92 Pinus 474.39 127.70 8.11 6.480 20.18

76 6 pine 120 Ha92 Pinus 492.91 177.62 8.28 7.670 7.32

77 7 pine 120 Ha92 Pinus 585.95 141.83 10.13 8.860 12.53

78 8 pine 120 Ha92 Pinus 630.35 53.79 10.66 9.710 8.94

79 9 pine 120 Ha92 Pinus 501.86 99.95 8.31 7.390 11.07

80 10 pine 120 Ha92 Pinus 575.20 147.08 9.77 8.560 12.38

81 1 pine 120 Ha87 Picea 465.19 176.12 7.91 6.620 16.33

82 2 pine 120 Ha87 Picea 497.79 173.82 8.51 8.030 5.65

83 3 pine 120 Ha87 Picea 499.76 99.63 8.38 7.540 9.98

84 4 pine 120 Ha87 Picea 456.92 147.79 7.60 6.340 16.63

85 5 pine 120 Ha87 Picea 478.22 89.68 8.21 7.320 10.89

86 6 pine 120 Ha87 Picea 499.00 99.25 8.59 8.040 6.32

87 7 pine 120 Ha87 Picea 464.17 96.77 7.82 6.720 14.05

88 8 pine 120 Ha87 Picea 565.29 138.27 9.32 8.770 5.97

89 9 pine 120 Ha87 Picea 570.70 142.34 9.74 9.210 5.48

90 10 pine 120 Ha87 Picea 573.67 118.97 9.70 8.380 13.55

91 1 pine 120 Pg91 Pinus 623.26 80.83 10.53 10.170 3.45

92 2 pine 120 Pg91 Pinus 578.05 145.16 9.88 8.720 11.76

93 3 pine 120 Pg91 Pinus 561.47 141.30 9.53 7.990 16.17

94 4 pine 120 Pg91 Pinus 597.19 135.23 9.75 8.850 9.26

95 5 pine 120 Pg91 Pinus 563.46 144.41 9.70 9.060 6.56

96 6 pine 120 Pg91 Pinus 503.90 180.07 8.73 7.510 14.06

97 7 pine 120 Pg91 Pinus 485.96 114.18 8.66 7.600 12.21

98 8 pine 120 Pg91 Pinus 485.94 159.09 8.33 7.220 13.28

99 9 pine 120 Pg91 Pinus 497.93 149.96 8.59 7.050 17.97

100 10 pine 120 Pg91 Pinus 574.99 111.23 9.71 8.110 16.52

101 1 spruce 60 Ib85 Pinus 530.11 122.63 9.05 8.330 8.00

102 2 spruce 60 Ib85 Pinus 530.18 113.35 9.21 8.430 8.45

103 3 spruce 60 Ib85 Pinus 513.49 129.30 8.75 8.110 7.35

104 4 spruce 60 Ib85 Pinus 533.54 109.49 9.25 8.520 7.84

105 5 spruce 60 Ib85 Pinus 473.85 147.11 8.21 7.490 8.73

106 6 spruce 60 Ib85 Pinus 462.85 132.75 8.02 7.090 11.63

107 7 spruce 60 Ib85 Pinus 514.45 136.40 8.67 8.160 5.95

108 8 spruce 60 Ib85 Pinus 548.69 126.17 9.53 9.040 5.16

109 9 spruce 60 Ib85 Pinus 485.09 86.25 8.42 7.500 10.99

110 10 spruce 60 Ib85 Pinus 507.92 122.25 8.85 8.080 8.68

111 1 spruce 60 Ib83 Picea 483.08 104.78 8.32 7.430 10.69

112 2 spruce 60 Ib83 Picea 482.97 145.47 8.40 7.810 7.04

113 3 spruce 60 Ib83 Picea 527.95 127.61 9.24 8.640 6.52

114 4 spruce 60 Ib83 Picea 495.50 112.17 8.51 7.840 7.83

115 5 spruce 60 Ib83 Picea 552.08 123.41 9.59 8.890 7.28

116 6 spruce 60 Ib83 Picea 524.48 135.64 9.11 8.470 7.04

117 7 spruce 60 Ib83 Picea 553.57 110.78 9.61 9.010 6.28

118 8 spruce 60 Ib83 Picea 514.31 130.08 8.96 8.280 7.62

119 9 spruce 60 Ib83 Picea 506.27 123.05 8.71 8.020 7.89

120 10 spruce 60 Ib83 Picea 536.48 119.34 9.31 8.600 7.60

121 1 spruce 60 Ha92 Pinus 503.67 73.83 8.76 8.130 7.12

122 2 spruce 60 Ha92 Pinus 510.81 75.67 8.86 8.050 9.09

123 3 spruce 60 Ha92 Pinus 498.68 82.72 8.68 8.110 6.52

124 4 spruce 60 Ha92 Pinus 511.40 76.52 8.81 7.990 9.33

125 5 spruce 60 Ha92 Pinus 516.35 68.81 8.99 8.370 6.86

126 6 spruce 60 Ha92 Pinus 465.65 99.34 8.11 7.450 8.21

127 7 spruce 60 Ha92 Pinus 452.93 174.34 7.86 7.370 6.26

128 8 spruce 60 Ha92 Pinus 504.02 67.92 8.76 8.270 5.57

129 9 spruce 60 Ha92 Pinus 477.09 114.97 8.25 7.460 9.56

130 10 spruce 60 Ha92 Pinus 485.32 76.21 8.23 7.590 7.83

131 1 spruce 60 Ha87 Picea 503.18 76.03 8.75 8.320 4.90

132 2 spruce 60 Ha87 Picea 535.55 74.40 9.28 8.700 6.33

133 3 spruce 60 Ha87 Picea 522.85 84.09 8.91 8.320 6.62

134 4 spruce 60 Ha87 Picea 566.06 57.99 9.75 9.180 5.85

135 5 spruce 60 Ha87 Picea 541.37 63.02 9.47 8.850 6.64

136 6 spruce 60 Ha87 Picea 490.14 82.94 8.51 7.920 6.88

137 7 spruce 60 Ha87 Picea 552.64 58.28 9.61 8.900 7.38

138 8 spruce 60 Ha87 Picea 490.75 57.95 8.55 8.090 5.34

139 9 spruce 60 Ha87 Picea 552.32 61.97 9.52 8.920 6.30

140 10 spruce 60 Ha87 Picea 555.01 55.51 9.66 9.070 6.11

141 1 spruce 60 Pg91 Pinus 502.58 81.33 8.53 8.000 6.22

142 2 spruce 60 Pg91 Pinus 505.26 76.45 8.75 8.220 6.05

143 3 spruce 60 Pg91 Pinus 539.91 64.88 9.36 8.900 4.91

144 4 spruce 60 Pg91 Pinus 517.66 85.46 8.97 8.370 6.70

145 5 spruce 60 Pg91 Pinus 547.85 62.35 9.40 8.870 5.64

146 6 spruce 60 Pg91 Pinus 529.39 66.76 9.26 8.710 6.00

147 7 spruce 60 Pg91 Pinus 526.80 84.64 9.18 8.620 6.16

148 8 spruce 60 Pg91 Pinus 534.93 76.62 9.24 8.740 5.38

149 9 spruce 60 Pg91 Pinus 501.69 86.70 8.69 8.130 6.48

150 10 spruce 60 Pg91 Pinus 513.73 87.81 8.94 8.410 5.98

151 1 spruce 120 Ib85 Pinus 538.16 61.88 9.36 7.568 19.15

152 2 spruce 120 Ib85 Pinus 528.47 149.87 9.21 8.225 10.72

153 3 spruce 120 Ib85 Pinus 532.72 132.43 9.24 8.006 13.35

154 4 spruce 120 Ib85 Pinus 528.52 141.55 9.19 8.041 12.49

155 5 spruce 120 Ib85 Pinus 484.52 124.20 8.41 6.735 19.96

156 6 spruce 120 Ib85 Pinus 482.99 127.37 8.40 6.837 18.57

157 7 spruce 120 Ib85 Pinus 495.40 120.86 8.57 7.043 17.85

158 8 spruce 120 Ib85 Pinus 508.35 113.20 8.80 7.260 17.50

159 9 spruce 120 Ib85 Pinus 487.42 125.84 8.44 6.880 18.50

160 10 spruce 120 Ib85 Pinus 500.31 121.23 8.72 7.008 19.59

161 1 spruce 120 Ib83 Picea 497.29 147.66 8.67 7.260 16.26

162 2 spruce 120 Ib83 Picea 511.92 140.19 8.99 7.420 17.45

163 3 spruce 120 Ib83 Picea 528.00 130.93 9.18 7.800 15.08

164 4 spruce 120 Ib83 Picea 492.39 158.35 8.65 7.080 18.10

165 5 spruce 120 Ib83 Picea 465.43 171.11 8.06 7.050 12.58

166 6 spruce 120 Ib83 Picea 528.50 125.18 9.11 7.380 18.98

167 7 spruce 120 Ib83 Picea 526.43 117.49 9.17 7.690 16.16

168 8 spruce 120 Ib83 Picea 489.44 151.38 8.54 6.830 20.01

169 9 spruce 120 Ib83 Picea 550.83 121.67 9.59 7.920 17.38

170 10 spruce 120 Ib83 Picea 554.37 113.76 9.61 7.820 18.61

171 1 spruce 120 Ha92 Pinus 482.83 97.00 8.37 7.210 13.89

172 2 spruce 120 Ha92 Pinus 546.65 74.31 9.47 8.090 14.58

173 3 spruce 120 Ha92 Pinus 521.57 87.93 9.05 7.580 16.26

174 4 spruce 120 Ha92 Pinus 558.49 82.65 9.59 8.220 14.27

175 5 spruce 120 Ha92 Pinus 508.15 74.24 8.88 7.850 11.57

176 6 spruce 120 Ha92 Pinus 509.94 106.79 8.85 7.420 16.15

177 7 spruce 120 Ha92 Pinus 525.74 83.81 9.14 8.030 12.18

178 8 spruce 120 Ha92 Pinus 532.76 83.00 9.27 7.960 14.17

179 9 spruce 120 Ha92 Pinus 501.81 75.39 8.77 7.520 14.28

180 10 spruce 120 Ha92 Pinus 489.79 90.52 8.55 7.240 15.37

181 1 spruce 120 Ha87 Picea 498.61 82.85 8.55 7.730 9.57

182 2 spruce 120 Ha87 Picea 536.23 81.65 9.36 8.330 11.08

183 3 spruce 120 Ha87 Picea 529.39 72.40 9.19 8.180 11.04

184 4 spruce 120 Ha87 Picea 489.09 80.95 8.37 7.310 12.66

185 5 spruce 120 Ha87 Picea 535.48 74.19 9.31 8.110 12.85

186 6 spruce 120 Ha87 Picea 541.63 67.84 9.39 8.400 10.58

187 7 spruce 120 Ha87 Picea 538.37 57.80 9.37 8.440 9.89

188 8 spruce 120 Ha87 Picea 477.36 85.66 8.31 7.340 11.73

189 9 spruce 120 Ha87 Picea 474.94 111.18 8.28 7.260 12.38

190 10 spruce 120 Ha87 Picea 495.98 85.65 8.63 7.560 12.43

191 1 spruce 120 Pg91 Pinus 548.02 97.16 9.57 8.570 10.46

192 2 spruce 120 Pg91 Pinus 484.39 99.55 8.51 7.610 10.55

193 3 spruce 120 Pg91 Pinus 545.12 86.89 9.49 8.600 9.39

194 4 spruce 120 Pg91 Pinus 495.92 105.48 8.53 7.510 11.95

195 5 spruce 120 Pg91 Pinus 518.59 98.93 8.99 8.070 10.18

196 6 spruce 120 Pg91 Pinus 497.82 118.36 8.68 7.640 11.99

197 7 spruce 120 Pg91 Pinus 540.14 87.54 9.38 8.490 9.55

198 8 spruce 120 Pg91 Pinus 510.77 111.75 8.66 7.600 12.25

199 9 spruce 120 Pg91 Pinus 537.73 92.35 9.23 8.350 9.59

200 10 spruce 120 Pg91 Pinus 486.39 118.41 8.45 7.490 11.42
